# Ultra-Micro-Scale-Fractionation (UMSF) as a Powerful Tool for Bioactive Molecules Discovery

**DOI:** 10.3390/molecules25163677

**Published:** 2020-08-12

**Authors:** Jason L. McCallum, Jennifer N. D. Vacon, Christopher W. Kirby

**Affiliations:** 1 Agriculture and Agri-Food Canada, Charlottetown Research and Development Centre, 440 University Avenue, Charlottetown, PE C1A 4N6, Canada; jvacon@upei.ca (J.N.D.V.); chris.kirby@canada.ca (C.W.K.); 2 University of Prince Edward Island, 550 University Avenue, Charlottetown, PE C1A 4P3, Canada

**Keywords:** UPLC-MS, bioactive molecules, microtiter plate assays, brine shrimp, *Artemia franciscana*, hops, *Humulus lupulus* L., *Cannabaceae*, lupulone, beta-acids

## Abstract

Herein is detailed the development and validation of an ultra-micro-scale-fractionation (UMSF) technique for the discovery of plant-based, bioactive molecules, coupling the advantages of ultra-performance liquid chromatography mass spectrometry (UPLC-MS) separations with microtiter plate-based bioassay screens. This novel one-step approach simultaneously uses UPLC to collect chemical profile information, while performing high-resolution fractionation, greatly improving workflow compared to methods relying on high-performance liquid chromatography (HPLC), solid phase extraction or flash systems for chromatographic separations. Using the UMSF technique, researchers are able to utilize smaller quantities of starting materials, reduce solvent consumption during fractionation, reduce laborious solvent dry down times, replace costly single-use solid-phase-extraction cartridges with reusable analytical-sale UPLC columns, reduce fractionation times to less than 10 min, while simultaneously generating chemical profile data of active fractions and enjoying superior chromatographic resolution. Using this technique, individual bioactive components can be readily purified, identified, and bioassayed in one step from crude extracts, thereby eliminating ambiguous synergistic effects often reported in plant-based natural products research. A successful case-study is presented illustrating the versatility of this technique in identifying lupulone as the principal cytotoxic component from hops (*Humulus lupulus* L.), using a brine shrimp (*Artemia franciscana*) model. These results confirm and expand upon previous cell-based bioassay studies using a more complex, multicellular organism, and add to our understanding of structure-function activity relationships for secondary metabolites in hops and the *Cannabaceae* plant family.

## 1. Introduction

Assay guided fractionation, a directed and iterative process of chemical extraction, purification, and bioassay, has arguably been the paradigm technique for pharmacognosists across decades of study. Indeed, the plant-derived chemotherapy agents paclitaxel [1], vinca alkaloids [2], and camptothecin [3], were all discovered using this approach, ultimately saving thousands of lives through applied clinical practice. Despite the rise and fall of synthetic combinatorial chemistry in pharmaceutical drug development [4], assay-guided fractionation has remained an indisputable tool in bioactive molecules discovery [5].

Initially, open-column chromatographic separations were predominantly utilized in assay guided fractionation, typically performed using a silica gel particle stationary phase. By today’s standards, these separations were largely inefficient, requiring copious volumes of volatile solvents and large amounts of starting materials (biological inputs), generally resulting in poorly resolved fractions, and seldom producing pure compounds. The incremental development of functionalized solid phases, including C-18 (octadecyl hydrocarbon chains), along with improved manufacturing techniques generating smaller and more uniform stationary phase particles, boosted chromatographic performance significantly; however, inefficient bioassays and low abundances of active compounds often still dictated large-scale extraction and purifications.

Combining solvent pumps to pressure-resistant columns led to the advent of flash-chromatography (FC), the first step towards efficient and automated separations, ultimately resulting in the development of computer-controlled high-performance liquid chromatography (HPLC) in the 1980s and ultra-performance liquid chromatography (UPLC/UHPLC) in the 2000s. As analytical platforms, both HPLC and UPLC have been widely adopted in natural products discovery laboratories, where minute quantities of extract (μg on column) could be efficiently and rapidly separated, and simultaneously chemically characterized (UV-Vis absorption, mass spectra). While HPLC has been amenable to the semi-preparative isolation of pure compounds in mg quantities, this process typically requires multiple rounds of repeated chromatographic isolation, spanning days of instrument time, and is therefore too laborious for the blind screening of individually purified compounds from crude extracts.

Somewhat paradoxically, despite the continued development of high-throughput and highly sensitive microtiter-based bioassay screens for a variety of biological models, FC, solid phase extraction (SPE) and open column methods remain the staple techniques for assay guided fractionation. While some research groups have developed and published successful HPLC-based fractionations in plant-based drug discovery, efforts primarily led by and recently reviewed by Matthias Hamburger [6], applications for UPLC-based ultra-micro-scale-fractionation (UMSF) in biological screening are only now emerging. Recent advances in micro-fluidics engineering, coupling UPLC instruments to robotics-controlled liquid-handlers (fraction collectors), offer promise as a powerful new approach to assay guided fractionation.

In the pursuit of streamlining the discovery of small-molecule phytochemical bioactives from plants, we have developed and optimized a novel UMSF technique coupling the power of ultra-performance liquid chromatography mass spectrometry (UPLC-MS) (Waters^®^ Acquity™ H-Class, Milford, MA, USA; TQD) with microtiter plate-based bioassays, using readily available, commercially-sourced components. Many natural products researchers are not sample limited, typically isolating multi-mg amounts of active compounds to conduct a full spectroscopic characterization and structural determination, which has presumably delayed the development and implementation of UMSF techniques; however, this approach presents several obvious advantages over conventional assay-guided fractionation methodologies, which are presented below.

The routine application of UMSF in plant-based small-molecule bioactives research has revolutionized our workflow, being potentially transformative in nature. Herein, we illustrate how UMSF workflow supplants the conventional assay approach, detail theoretical considerations underlying the technique, make cost comparisons with traditional assay-guided fractionation strategies, and present case-study data illustrating the utility of the method.

## 2. Results and Discussion

### 2.1. The UMSF Approach

**Figure 1:**
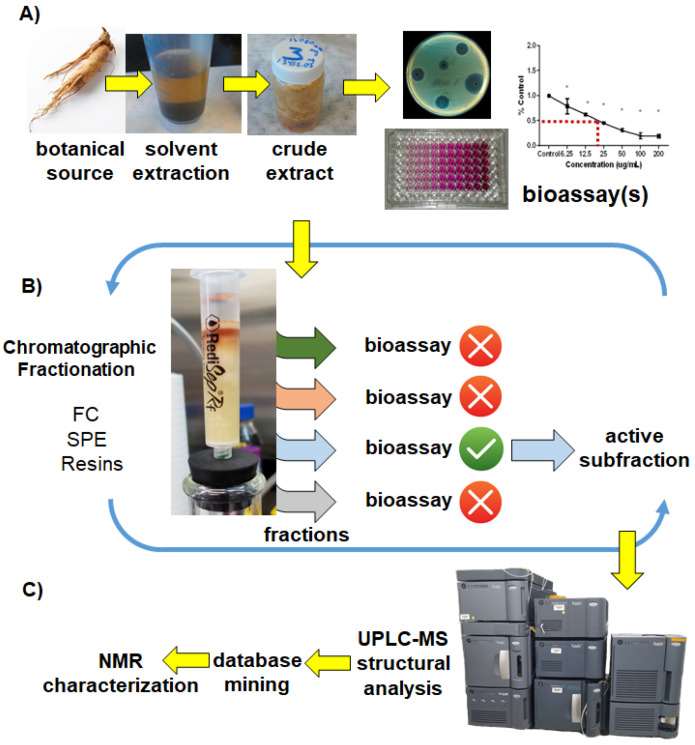
Classic assay-guided fractionation model. (**A**) Crude extracts are prepared, bioassayed, and triaged for importance; (**B**) bioactive crudes are chromatographically fractionated into simpler mixtures. Each fraction is bioassayed and further purified through iterative rounds of chromatography; (**C**) active compounds are identified and characterized via analytical platforms including ultra-performance liquid chromatography mass spectrometry (UPLC-MS), database queries and nuclear magnetic resonance (NMR) structural elucidation.

Conventional assay-guided fractionation schemes rely on an iterative process of bioassay (Figure 1A) and chromatographic fractionation (Figure 1B), typically relying on low pressure FC, vacuum driven SPE cartridges, or other resins (size exclusion, ion-exchange etc.), eventually followed up by semi-preparative HPLC purification. Numerous rounds of bioassays, coupled to increasingly complex chromatographic purifications are required to follow biological activity down to the level of individually pure compounds, which are further characterized via a suite of analytical techniques (Figure 1C). Initially this “classic” approach was quite successful in the characterization of new biologically active chemical agents from plants, microbes, and marine organisms, heralding a golden age of natural products drug discovery and resulting in the development of various therapeutics, including antibiotics, statins, and anti-cancer chemotherapy agents [7]. However, the frequent “rediscovery” of known compounds after such a laborious and time-consuming process, one that often generates large volumes of waste solvents due to lengthy and inefficient chromatographic separations, and which requires rather large (kg+) quantities of starting biological inputs, has contributed to a gradual decline of the technique in recent decades.

More recently, chemical dereplication, essentially a pre-screening of crude extracts using a combination of high-resolution-mass-spectrometry (HR-MS), tandem mass spectrometry (MS/MS), and UV-Vis spectroscopy coupled to natural products database queries and metabolomics-style data processing, has been enlisted to assist in the discovery of new chemical scaffolds, and thereby avoid the rediscovery of known molecules [8,9,10]. However, while chemical dereplication allows researchers to focus their efforts on the discovery of new chemical compounds, it often fails to identify and attribute any useful biological activity to these novel molecules. As the isolation and structural elucidation of novel natural products has become common place, the identification of useful biological/therapeutic targets for said molecules has instead become a limiting bottle neck for scientific publication and intellectual property development.

Conversely, while complex crude mixtures from botanical sources often possess strong biological activity in initial bioactivity screens, this bioactivity is frequently and counter-intuitively lost during the subsequent purification and analyte concentration steps; ultimately resulting in various arguments about chemical synergism and complex (multiple) modes of action [11,12,13]. While these arguments may satisfy natural-health, ethnobotanical, and traditional-medicine practitioners and ultimately add to our understanding of chemical ecology, they preclude the development of modern day pharmaceutical agents, for which a direct, dose-dependent response for purified molecules and their individual biological effects is usually required.

**Figure 2:**
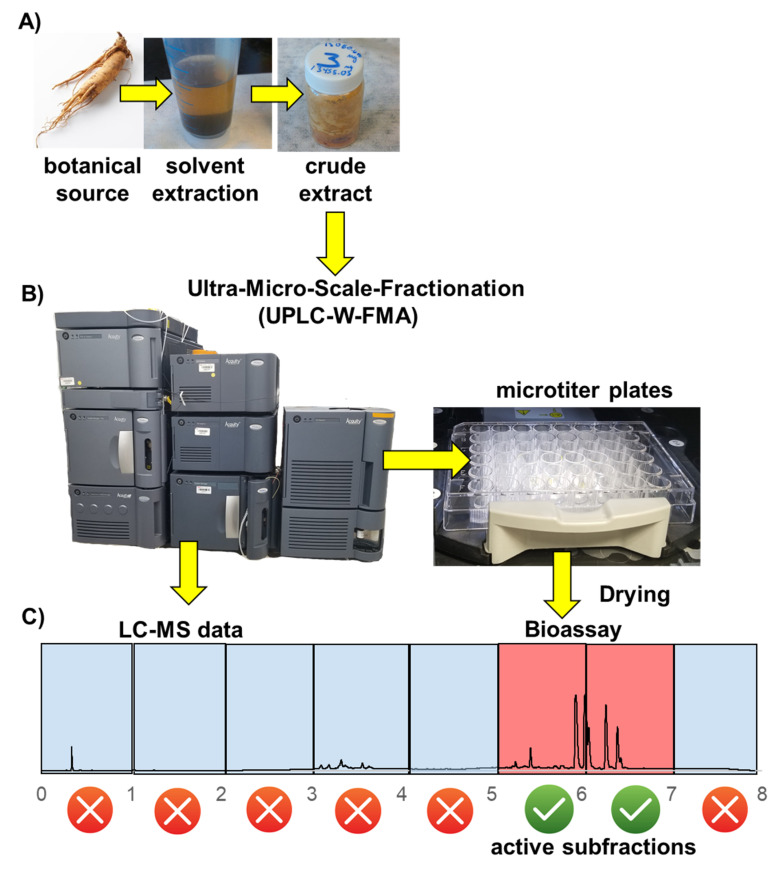
Ultra-micro-scale-fractionation (UMSF). (**A**) Crude extracts are prepared; (**B**) crude extracts are simultaneously analyzed and fractionated into simpler mixtures using high-resolution ultra-performance liquid chromatography (UPLC) separations. Individual fractions are collected into wells of microtiter plates for later bioassay; (**C**) chemical characteristics of active fractions are queried from existent liquid chromatography mass spectrometry (LC-MS) data, to identify and triage lead compounds.

The novel UMSF technique (Figure 2) relies on advances in UPLC-MS achieved in the past decade, which offer increased chromatographic performance, shorter run times, less solvent consumption, and smaller injection sizes compared to HPLC, in order to greatly streamline the laborious chromatographic fractionation and bioactivity testing stages associated with assay guided fractionation. Essential to the development of the UMSF technique was the commercial release of an analytical scale fraction manager (W-FMA), initially marketed for the isolation of pure compounds using UPLC systems. The W-FMA module is a software-controlled, robotic fraction collector, designed to handle the very narrow peaks associated with UPLC separations, with corresponding small internal dead volumes, fast switching valve actuators, make-up solvent flow for improved needle cleaning, and subsequently minimizes sample carry over between fractions. In the UMSF approach, fractionation is conducted using an analytical UPLC column (Figure 2B), resulting in highly efficient and reproducible chromatographic separations, while simultaneously collecting UV-Vis absorption and/or MS data on the resultant fractions (Figure 2C). To our knowledge, we are the only laboratory in Canada equipped with a W-FMA module, and are certainly among the pioneers developing its use as a bioactivity testing pipeline.

**Figure 3:**
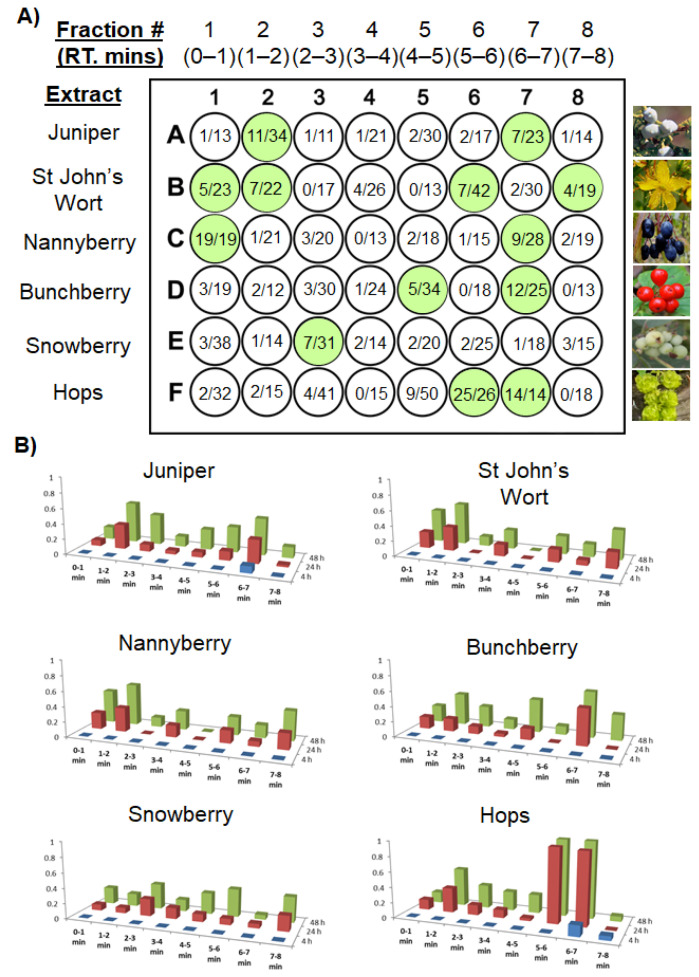
Representative bioassay screening data generated using UMSF technique. (**A**) Raw, individualized brine shrimp mortality data, taken at 24 h post exposure, highlighting active fractions; (**B**) averaged brine shrimp mortality data (n = 3), presented across time for UMSF derived samples; blue = 4 h exposure; red = 24 h exposure; green = 48 h exposure.

**Figure 4:**
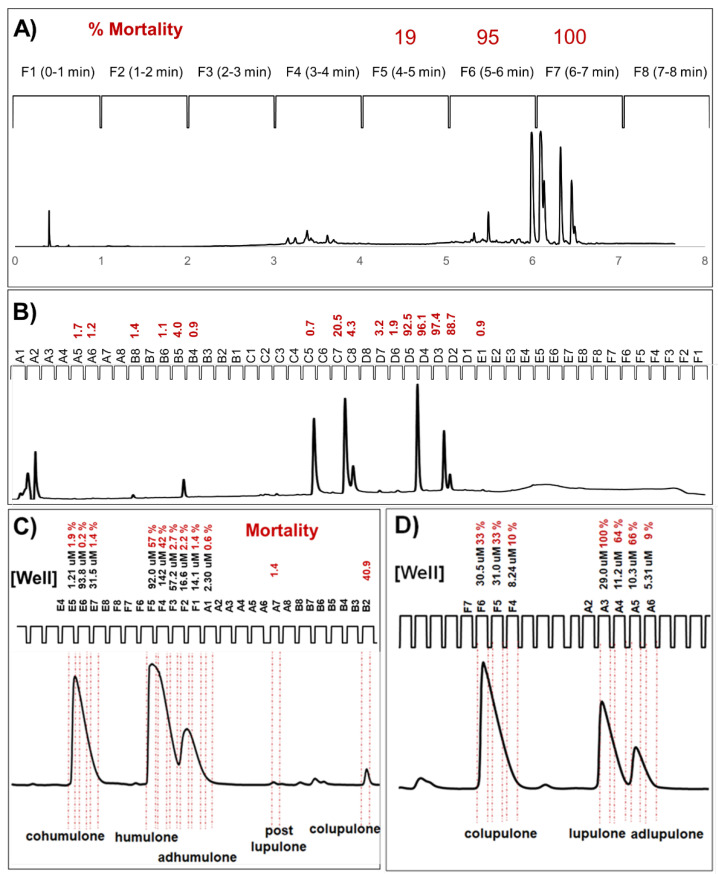
Representative bioassay screening data generated using UMSF technique. (**A**) Initial screening data for *Humulus lupulus* L. extract, with mortality assessed at 24 h post exposure using low-resolution 1 min wide retention windows; (**B)** optimized, higher-resolution 10 s retention time windows; (**C**) fully optimized, 5 s wide retention time windows, showing pure alpha acid compounds in individual wells; (**D**) fully optimized, 5 s wide retention time windows showing pure beta acid compounds in individual wells. Chromatograms monitored at 250 nm.

Using MassLynx™ software (Waters, Milford, MA, USA), the W-FMA collects UMSF by preprogrammed retention time windows. In preliminary screens of crude botanical extracts these windows are typically assigned a 1 min duration, and are collected into sterile 48-well tissue-culture microtiter plates most amenable to our specific downstream bioactivity screening, a brine shrimp (*Artemia franciscana*) lethality bioassay model for the identification of potential cytotoxic molecules (Figure 3). Solvents are subsequently dried through centrifugal evaporation and/or lyophilizing, prior to conducting bioassays. Initial screening of crude botanical extracts is performed using a generic 8 min reverse-phase chromatographic separation, with a single 48 well microtiter plate holding fractions from six distinct biological inputs (Figure 3A). Using this approach, roughly one plate of fractions is generated per hour of instrument time, and multiple plates from a single day’s throughput are dried overnight. Crude data, detailing the number of dead shrimp per well (Figure 3A), can be followed across time (4 h, 24 h, 48 h), in triplicate, and processed in a variety of ways (Figure 3B). Once leading bioactive fractions are identified from this initial screening (Figure 3A,B), solvent gradients and collection time windows can be optimized to improve chromatographic resolution among peaks, to the point of having pure compounds in individual wells (Figure 4C,D). Compared to traditional fractionation techniques, which rely on FC or SPE cartridges, the UMSF technique offers highly reproducible and superior chromatographic performance, consumes minimal amounts of solvents, produces minimal amounts of waste, and reuses the same analytical sub 2 μm particle size column for thousands of injections, compared to single-use FC or SPE cartridges.

Most bioactives discovery programs already have dedicated UPLC/UHPLC analytical chemistry platforms in place for both quantitative and qualitative analyses, be it the untargeted profiling of crude extracts or fractions prepared thereof, or purity checks for isolated peaks, however, most groups still rely on inefficient FC or SPE to conduct their initial fractionation. Adaption of the UMSF technique can be achieved through a modest investment into a fraction collector while boosting throughput and reducing consumable costs.

### 2.2. Theoretical Considerations Underlying the UMSF Technique

Dried botanical tissues generally contain at least 1% (by mass) small molecule extractables, and thereby 1 g of source input will typically yield a minimum of 10 mg of dried crude extract, more than sufficient for UMSF screening. Using such small starting masses greatly reduces the volume of solvents required for downstream extractions, thereby limiting drying down steps and the generation of hazardous wastes. Additionally, using small amounts of starting materials also limits any potentially damaging effects from overharvesting of wild-growing botanical resources.

A typical crude extract from botanical sources contains on the order of 100 individual small-molecule components, with most extracts soluble at 15 mg/mL or greater in a suitable solvent system (aqueous blends of methanol, ethanol, isopropanol). A typical maximum UPLC injection size is 10 μL, resulting in 150 μg of crude material delivered to the analytical column, given a stock concentration of 15 mg/mL. Within a given crude extract, some compounds are much more abundant than others, while others are present only in trace amounts, but overall, one can assume an average of approximately 1.5 μg of each individual compound that is delivered to the column. As these small molecules elute from the column, they are captured in wells of microtiter plates. The 48-well cell-culture microtiter plate commonly used in our laboratory has a working volume of 350 μL in downstream bioassays, resulting in 1.5 μg of given molecule **X** in 350 μL of media. As most bioactive molecules have molecular weights less than 500 Da [14], one can conclude the final molar concentration for most individual molecules in the mixture is around 8.6 μM. Drug-like molecules should be biologically active at single digit μM or lower ranges, thus, this UMSF screening system will identify bioactive molecules, at physiologically relevant concentrations, using analytical UPLC scale injection sizes.

With regard to the aforementioned assumptions regarding the number of individual components present in the extract, their overall abundances, or their molecular weights, a 2-fold error in any two factors, in either direction, would yield biologically active molecules ranging from 2.1 μM to 34.2 μM (in-well): still ideal for assay-guided screening efforts.

### 2.3. Cost Analysis—UMSF Is Friendly on Your Budget and the Environment

#### 2.3.1. Capital Investment

The initial purchase price of a UPLC fraction collector (W-FMA) module is comparable to procuring a flash LC system. Many natural products and drug discovery labs already have purchased UPLC analytical platforms, and the W-FMA module is comparable to adding a new detector to the system.

#### 2.3.2. Consumables

Columns: in our laboratories, a single analytical UPLC column (~USD 1200) can be reused thousands of times with proper care, thereby averaging less than USD 1/sample. Conventional FC relies on single-use silica, or limited reuse C-18 columns, averaging USD 10–20 per sample. SPE setups similarly rely on single-use cartridges averaging USD 5–20 per sample, depending on the mass of sorbent. For research programs fractionating and screening hundreds of biological inputs per year, the cost savings associated with using a UPLC column can be substantial.

Solvents and Drying: the UMSF approach uses ~5 mL of solvent per sample run, roughly half of which is water. FC methods typically use 1–2 L of solvent per run, while SPE methods still need 100 s of mLs. The reduced volumes of solvents associated with UMSF requires shorter downstream drying times and produce less chemical waste for disposal. Microtiter plates can be dried quickly in parallel, while most FC and SPE fractions are collected into test tubes, scintillation vials, or round bottom flasks, which typically require lengthy rotary evaporation steps, generally one sample at a time. Microtiter plates are low cost, with many samples fractionated into one plate. While disposable glass tubes and scintillation vials are also low cost, a single FC or SPE fractionation typically requires dozens of such vessels, producing large volumes of landfill waste.

Biological Starting Materials: UMSF sample injections are small (10 μL/150 μg), thereby typically requiring less than 1 g of starting materials for extraction. FC and SPE fractionation techniques generally load 100 s of mg on-column, thereby sometimes requiring kg amounts of starting materials. As many natural products researchers screen a wide variety of biological inputs, preparing initial crude extracts is a time-consuming, bottleneck step. Minimizing the amounts of biological materials being collected, dried, stored, and ultimately extracted, is paramount to improving workflow, and UMSF allows for successful fractionation and identification of biological activities from sub-gram masses of starting materials; a single leaf, fruit, or flower is often enough. This reduces harmful effects associated with overharvesting wild-collected materials, requires less storage space for dried botanical source materials, while reducing both the volumes of solvents used and dry down times associated in generating crude extracts.

## 3. Illustrative Case Study—Lupulone Beta Acids from Hops Are Bioactive against Brine Shrimp

Using the UMSF technique presented above **(Figure 2**), we produced and screened extracts from a variety of botanical source inputs including Rocky Mountain Juniper berries (*Juniperus scopulorum*), St. John’s Wort flowers (*Hypericum perforatum*), Nannyberry fruits (*Viburnum lentago*), Bunchberry Dogwood fruits (*Cornus canadensis*), Western Snowberry fruits (*Symphoricarpos occidentalis* Hook), and Hops flowers (*Humulus lupulus*) (Figure 3A,B). Crude extracts were prepared using a modified QuEChERs (acetonitrile-salting out) procedure, omitting the secondary dispersive solid-phase sorbent clean up steps [15]. The resulting QuEChERs acetonitrile fractions were dried down in parallel via a nitrogen stream, desiccated overnight, and were resuspended in water:methanol (1:1) at 15 mg/mL.

A 10 μL aliquot of each crude fraction was separated according to a generic 8 min long UPLC-DAD-FMA method, with 1 min wide retention time windows used to collect each fraction (Figure 3A). The initial generic 8-min gradient was developed as a blind fractionation method, for extracts of unknown chemical composition containing a mixture of both high- and low-polarity compounds. In this initial screening method, 1 min wide retention time intervals were collected for preliminary fractionation and screening, simply because the 48 well microtiter plates (6 × 8) have 8 wells per row and a suitable generic chromatographic method was conveniently 8 min long. The lethality of these fractions on brine shrimp (*Artemia franciscana*) was evaluated in triplicate, at 4 h, 24 h and 48 h exposure, with raw results recorded as the number of dead shrimp per well population (Figure 3A), and converted to a mortality measurement ranging from no deaths (0) to 100% mortality (1) (Figure 3B). Exceptionally strong brine shrimp toxicity was observed for individual fractions in Nannyberry (well C1) and Hops (wells F6, F7), yielding leading candidates for further characterization. Examining the UV-Vis and MS chromatographic profiles generated during the preliminary fractionation process (Figure 2C, Figure 4A and Supplemental File 1), a variety of prenylchalcone, alpha and beta acid derivatives were identified in hops fractions F6 and F7, along with trace amounts of oxidized alpha and beta derivatives (Table 1A); all these compounds having been commonly reported from this botanical source [16,17,18,19,20,21,22,23,24].

As the initial 1 min screening windows generated a complex mixture of compounds in hops fractions F6 and F7 (Figure 2C and Figure 4A; Table 1A), a higher resolution fractionation and screening method utilizing an optimized chromatographic gradient and a shorter 10 s retention time collection window were used to independently evaluate the individual effects of prenylchalcones: wells A8 → B4; alpha acids: wells C5 → C8; and beta acids: wells D7 → D2 (Figure 4B; Table 1B). Chromatography was further optimized for alpha acids (Figure 4C; Table 1C) or beta acids (Figure 4D; Table 1D), using extracts enriched for alpha or beta acids respectively, this time using 5 s retention time collection intervals, to evaluate the effects of pure compounds on brine shrimp. Alpha acids were fractionated to purity into individual wells: cohumulone = E5 →E7; humulone = F5 → F3; adhumulone = F2 → A1 (Figure 4C; Table 1C), while beta acids were fractionated to purity into individual wells: colupulone = F6 → F4; lupulone = A3 →A4; adlupulone = A5 →A6 (Figure 4D; Table 1D). The speed and ease of developing new chromatographic methods using the UPLC platform greatly improved the throughput and optimization of chromatography, resulting in pure compounds being individually bioassayed. Obtaining pure compounds using conventional assay guided fractionation techniques could be expected to take weeks of method development, scaled up extraction/isolation, and iterative rounds of labor-intensive chromatography, compared to a single day of optimization and collection using the UMSF technique and a UPLC platform.

Individual molar concentrations for pure compounds in each bioassay well could be calculated from integrated areas under the curve, using linear regression lines generated from ICE-3 primary reference standard calibration curves (Figure 4C,D; Table 1C,D) [23]. Based on the bioassay results from individual wells containing pure compounds (Figure 4C,D; Table 1C,D), it is clear the hops beta acids including colupulone, lupulone, adlupuluone, and postlupulone are the principal toxic molecules vs. brine shrimp. Lupulone and adlupulone display LC_50_ values less than 10 μM at 24 h, a dose roughly one order of magnitude lower than their alpha acid counterparts (Figure 4C,D; Table 1C,D). Amongst the individual beta acids, lupulone and adlupulone are more toxic than colupulone; this trend also extends to comparisons of individual toxicities amongst the alpha acids, where cohumulone is the least toxic. The side chains for colupulone and cohumulone originate from a valine-derived isobutyric-CoA, while the side chain of the n- and ad- variants are one CH_2_ group longer, having come from leucine and isoleucine substrates [25]. How this slight chemical difference translates into increased toxicity remains speculative, but longer and more highly branched aliphatic side chains are associated with increased receptor-ligand signalling potency amongst various cannabinoids [26,27], the closest biochemical homologue to hops bitter acids. While the toxicity of hops bitter acids versus gram positive bacteria is believed to involve the disruption of cellular homeostasis, with beta acids acting as especially efficient membrane-soluble ionophores [22], it is unclear whether the slight overall polarity differences presented by lupulone and adlupulone compared to colupulone can explain our observations; we speculate that brine shrimp mortality is caused by receptor mediated mechanisms as opposed to ion-leakage across the cell membrane. Further investigations into structure-function relationships amongst individual hops compounds using more robust quantitative methods, serial dilution dose-response curves, and including calculations of LC_50_ values is warranted and ongoing in our laboratory.

Interestingly, when the hops alpha acids were screened as purified compounds (Figure 4C; Table 1C), their toxic effects on brine shrimp were greatly reduced, compared to being coadministered as a mixture with prenylchalcones in the lower-resolution preliminary screening (Figure 2C and Figure 3A—hops fraction F6; Table 1A), illustrating how chemical synergism can be detected in complex mixtures and subsequently lost during iterative rounds of compound purification. Using UMSF to quickly obtain pure compounds and thereby eliminate chemical synergism from confounding bioactivity screening assays allows for the attribution of biological activity to single molecules, greatly facilitating the drug discovery process.

The cytotoxic effects of hops beta acids (lupulone derivatives) have already been well established using a variety of human cancer cell screening assays, including SK-MES lung [28], MDA-MB-231 and MCF-7 breast [28,29], SW 620 colon [30], along with PC3 and HT29 prostate lines [31], with various lupulone derivatives showing LC_50_ values in the single digit micromolar range. In these studies, structure function relationships amongst lupulones (co- vs. n- vs. ad- vs. post-) show similar trends to our results, with colupulone showing lower activity than n-lupulone. Similarly, alpha acids were shown to be less cytotoxic than beta acid derivatives by at least a factor of two [28,31]. These results have interesting chemical ecology implications and warrant further investigation.

Using the UMSF approach, we successfully attributed potent bioactivity to individual phytochemical components arising from a complex botanical extract, using a brine shrimp toxicity model commonly employed in high-throughput cytotoxicity screening [32,33,34]. The brine shrimp lethality bioassay adapted in our laboratory is robust, effective, and low-tech, needing little more than a stereomicroscope and multichannel pipettor, and not requiring laminar flow hoods, sterile clean rooms, cell-incubators, nor fluorescent reagents or plate readers. Juvenile brine shrimp progress through rapid morphological developmental queues in their first few days post-hatching, a process involving multiple rounds of DNA replication and cell divisions. Any exogenously applied chemical substances which interfere with cell-cycle regulation, DNA synthesis, replication or mitosis will be effective at halting their maturation and development, and thereby kill the nauplii, making them potentially attractive cytotoxic agents versus cancer cells that undergo similar rapid cell divisions and DNA replication. Additionally, being invertebrate arthropods, brine shrimp are a convenient model system for evaluating potential anti-insect activity of plant secondary metabolites. Captive insect rearing and toxicity screening is often an intensive and difficult process, while brine shrimp are a simpler model system displaying some homologous biochemistry. Identifying plant secondary metabolites toxic to brine shrimp suggests potential ecological functions versus insect herbivores, allowing researchers to target specific molecules in follow up studies. While these specific hops compounds have been previously identified as anti-cancer agents in cell-based assays, this manuscript confirms their effectiveness in a multi-cellular organism and demonstrates the utility of the novel UMSF approach in identifying bioactive molecules as a first step in high throughput screening efforts. The UMSF approach allows for rapid optimization of chromatography and generates useful bioassay results from μg quantities of pure compounds, ultimately eliminating many laborious and costly wet-chemistry and isolation methods normally required in assay guided fractionation. Using this UMSF approach, novel biological activity has been identified in a variety of botanical extracts not presented herein, being attributed to small-molecules whose nuclear magnetic resonance (NMR) structural characterization is ongoing in our laboratories. Further development and refinement of additional high-throughput bioassay screens using the UMSF approach is also ongoing, including anti-fungal and anti-bacterial activities, enzyme inhibition studies, and allelopathic seed-germination assays. We believe the potential utility of this novel UMSF technique in pre-clinical drug discovery could ultimately prove to be transformative in nature, greatly increasing workflow efficiency, reducing input costs, benefiting the environment, and leading to reuptake of the assay-guided fractionation process for screening natural products.

## 4. Materials and Methods

### 4.1. Chemicals and Consumables

All chromatographic solvents were liquid-chromatography mass spectrometry (LC-MS) Optima™ grade (Thermo Fisher Scientific, Nepean, ON, Canada). SupraPur™ formic acid (98–100%), ACS grade acetonitrile, OmniSolv^®^ methanol, 48-well cell culture plates (non-surface treated), and QuEChERS salts (magnesium sulfate, trisodium citrate dehydrate, sodium chloride) were from VWR International (Mississauga, ON, Canada). Individual hops compounds were quantitated using linear regression calibration curves based on 7-point serial dilutions of ICE-3 (International Calibration Extract), a certified reference material commonly utilized by brewing chemists (Labor-Veritas AG, Zürich, CH, Switzerland). Absolute ethanol was from Commercial Alcohols (Greenfield Global, Boucherville, QC, Canada).

### 4.2. Plant Materials

Lyophilized or air dried plant materials including Rocky Mountain Juniper berry (*Juniperus scopulorum* Hook), St. John’s Wort flowers (*Hypericum perforatum*), Nannyberry fruits (*Viburnum lentago*), Bunchberry Dogwood fruits (*Cornus canadensis*), Western Snowberry fruits (*Symphoricarpos occidentalis*), and Hops cones (inflorescences) (*Humulus lupulus*) were used in this demonstrative pilot study. Approximately 2 g of dried materials were cryogenically frozen in liquid nitrogen, ground to a fine powder via mortar and pestle, homogenized, and 0.4 g aliquots used to prepare crude extracts. Rocky Mountain Juniper, Nannyberry, and Snowberry fruits were identified and harvested from AAFC research plots at the Agroforestry Development Centre, Indian Head, Saskatchewan, Canada, by Research Biologist William Schroeder. St. John’s Wort flowers, and Bunchberry Dogwood fruits were identified and wild-harvested from the Charlottetown area of Prince Edward Island, Canada, by Dr. Jason McCallum. Hops cones were of the cultivar ‘Cascade’ grown at AAFC’s experimental hopyard in Harrington, Prince Edward Island, Canada, by Dr. Aaron Mills.

### 4.3. Extraction

A modified QuEChERs (acetonitrile-salting out) procedure, omitting the secondary dispersive solid-phase sorbent clean-up steps was used to prepare extracts [15]. In brief, 400 mg of cryogenically ground samples were extracted with 20 mL of acetonitrile:water (3:1), in 40 mL glass scintillation vials, in a sonic bath at 37 °C, for 1 h. Post-extraction, QuEChERs salts (2 g magnesium sulfate; 0.7 g trisodium citrate dihydrate; 0.3 g sodium chloride) were added to the resultant slurries, vortexed vigorously for 30 s, and centrifuged (10 min, 1000× *g*) to promote phase separation. 10 mL of the upper acetonitrile layer was transferred to pre-weighed 20 mL scintillation vials and dried via a gentle stream of nitrogen, prior to overnight desiccation under vacuum. The resultant crude extracts were resuspended at 15 mg/mL in water:methanol (1:1), prior to separation and fractionation by UPLC-PDA-WFMA.

### 4.4. Chromatographic Separation and Fractionation via UPLC-PDA-FMA-TQD

Samples were separated, fractionated and analyzed using a Waters Acquity™ H-Class UPLC™ equipped with FTN (flow through needle) autosampler assembly, eλ PDA (photodiode array), W-FMA (fraction manager analytical) and TQD-MS (tandem quadrupole mass spectrometer) (Waters Corporation, Milford, MA, USA), running MassLynx™ 4.1 software. Separations were performed using a CSH™-C18 column (100 mm × 2.1 mm; 1.7 μm particle size), equipped with CSH™-C18 VanGuard™ pre-column (5 mm × 2.1 mm; 1.7 μm particle size, Waters, Milford, MA, USA), held at 45 °C. Prior to injection (10 μL), samples were thermostatically maintained at 25 °C to prevent precipitation, and all mobile phases were filtered and degassed using an in-line integrated system. All chromatographic methods utilized a three-solvent blend, where solvent A = water; solvent B = acetonitrile; solvent C = 10% formic acid in water.

### 4.5. Initial Screening: (1 min Retention Time Windows—Figures 3 and 4A; Table 1A)

The following generic screening gradient was employed at 0.600 mL/min, showing good chromatographic separation amongst polar, intermediate and non-polar components: t(0) A:B:C = 90:5:5; t(1 min) A:B:C = 90:5:5 (isocratic); t(4 min) A:B:C = 50:45:5; t(6 min) A:B:C = 0:95:5; t(8 min) A:B:C = 0:95:5 (isocratic); followed by a 2 min post-run re-equilibration period at A:B:C = 90:5:5. The W-FMA module was set to collect fractions in 1 min intervals (F1 = 0–1 min; F2 = 1–2 min; F3 = 2–3 min; F4 = 3–4 min; F5 = 4–5 min; F6 = 5–6 min; F7 = 6–7 min; F8 = 7–8 min), with individual fractions collected into 48-well cell culture plates. Using this fractionation strategy, each 48-well cell culture plate can accommodate 6 independent fractionation runs of 8 wells each.

### 4.6. Hops-Specific Chromatography: (10 s Retention Time Windows—Figure 4B; Table 1B)

The following gradient was employed at 0.600 mL/min, showing improved chromatographic separation amongst hops bitter acid components: t(0) A:B:C = 45:50:5; t(5.5 min) A:B:C = 0:95:5; t(6.75 min) A:B:C = 0:95:5 (isocratic); t(7 min) A:B:C = 45:50:5; t(8 min) A:B:C = 45:50:5 (isocratic). Samples were fractionated into 10 s retention time windows, with one injection resulting in filling all 48 wells of a single cell culture plate.

### 4.7. High-Resolution Screening: (5 s Retention Time Windows—Figure 4C,D; Table 1C,D)

Alpha acid (humulone derivative) separations were optimized using the following gradient at 0.500 mL per minute: t(0) A:B:C = 30:65:5; t(1 min) A:B:C = 30:65:5 (isocratic); t(4 min) A:B:C = 15:80:5; t(4.75 min) A:B:C = 0:95:5; t(6.75 min) A:B:C = 0:95:5 (isocratic); t (7 min) A:B:C = 30:65:5; t (8 min) A:B:C = 30:65:5 (isocratic). Beta acid (lupulone derivative) separations were optimized using the following gradient at 0.600 mL/min: t(0) A:B:C = 30:65:5; t(1 min) A:B:C = 30:65:5 (isocratic); t(4 min) A:B:C = 15:80:5; t(4.75 min) A:B:C = 0:95:5; t(6.75 min) A:B:C = 0:95:5 (isocratic); t(7 min) A:B:C = 30:65:5; t(8 min) A:B:C = 30:65:5 (isocratic). Samples were fractionated into 5 s retention time windows, with one injection resulting in filling all the wells of two 48 well plates.

For all UPLC chromatographic isolations, total diode array scans (240–600 nm) were collected at 20 Hz. After each microtiter plate was filled, the W-FMA module was taken offline using configuration settings, with samples being reinjected, separated using the same chromatographic method, and analyzed using the TQD detector in series after the UPLC, providing MS information to accompany the previously generated PDA data. Electrospray ionization (ESI) scans (150–1500 amu) were simultaneously collected in both positive and negative modes, where the capillary voltage was 3.5 kV (+ve) or −2.7 kV (−ve); source temperature 150 °C; desolvation gas (N2) temperature 400 °C; desolvation gas flow 850 L/h; cone gas flow 50 L/h; cone voltage 60 V (+ve) or −37 V (−ve); extractor 5 V (+ve) or −3 V (−ve); RF lens 1.9 (+ve) or −2.3 (−ve); and collision gas (Ar) flow of 0.35 mL/min.

For each set of experiments, fractionation and bioassays were performed in triplicate, with multiple negative control runs consisting of 10 μL methanol injections, to account for possible chemical interferences arising from UPLC system leachates or components from the tissue culture plates themselves. Fractionated samples in cell culture plates were dried overnight using an acid-resistant Labconco™ Centri-vap system (Kansas City, MO, USA). Post-drying, residues in individual wells were resuspended in 20 μL of water:ethanol (1:1), immediately prior to preparation of bioassay plates, with negative controls also receiving water:ethanol (1:1) aliquots to assess the possible harmful effects of ethanol.

### 4.8. Biological Assays

An in-house brine shrimp cytotoxicity screen was developed and optimized, as per previously published methods and manufacturer’s instructions. Briefly, a saline solution was made by dissolving 36 g of Instant Ocean^®^ marine salts (Marineland, Blacksburg, VA, USA) per 100 mL of Milli-Q water. Brine shrimp cysts (*Artemia franciscana*) (Ocean Star International, Snowville, UT, USA) were incubated at 0.1 g per 100 mL of saline solution in Erlenmeyer flasks, with gentle aeration, and heating (27 °C) overnight. After 16 h of incubation, newly hatched shrimp nauplii were collected using Pasteur pipette, a process greatly facilitated by their attraction to and schooling in response to an exogenous light source, prior to bioassay plating. Each tissue culture well contained a total of 20 μL of water:ethanol (1:1), diluted with 300 μL saline solution, and 2 drops of nauplii (~30 μL; 15–40 shrimp per well), resulting in a final volume of ~350 μL (±10 μL), and a final ethanol concentration of less than 3% (v/v), demonstrated to be non-lethal to brine shrimp. Nauplii were observed under a low power stereomicroscope (~30x magnification) (Motic SMZ-168, Motic Instruments Inc., Richmond, BC, Canada), with status assessed at 4, 24 and 48 h post-exposure, and the number of dead shrimp per well recorded. Results from negative controls were calculated and subtracted from experimental wells, resulting in adjusted mortality values.

## Figures and Tables

**Table 1:** Principal bioactive compounds from Hops (*Humulus lupulus* L.) identified through UMSF. (**A**) Low-resolution 1 min retention time windows; (**B**) medium-resolution 10 s retention time windows; high-resolution 5 s retention time windows (pure compounds) from: (**C**) humulone-enriched extract; (**D**) lupulone-enriched extract. All mortality data were collected 24 h post-exposure in an *Artemia franciscana* toxicity assay (n = 3).

**(A) Low-resolution 1 min retention time windows**							
**Fraction**	**% Mortality**	**Principal Components**		**Minor Components**		Trace Components *	
F6 (5–6 min)	95	cohumulone, xanthohumol		desmethylxanthohumol, posthumulone		oxidized alpha acids, oxidized iso-alpha acids	
F7 (6–7 min)	100	cohumulone, humulone, adhumulone, colupulone, lupulone, adlupulone		prehumulone, adprehumulone, postlupulone, prelupulone, adprelupulone		hydroxytricyclolupones	
**(B) Medium-resolution 10 s retention time windows**							
**Fraction**	**% Mortality**	**Principal Components**		**Obs. MW (Da)**	**ESI-NIM (Intensity)**	**Deprotonated Ion**	**UV-Vis λmax (nm)**
B8	1.4	desmethylxanthohumol		340	339(100)	[M − H]^−^	366
B6	1.1	xanthohumol		354	353(100)	[M − H]^−^	369
B5	4.0	xanthohumol		354	353(100)	[M − H]^−^	369
B4	0.9	xanthohumol		354	353(100)	[M − H]^−^	369
C5	0.7	cohumulone		348	347(100)/278(40)	[M − H]^−/^[M-C_5_H_9_ − H]^−^	285/330(sh)/360(sh)
C7	20.5	humulone		362	361(100)/292(40)	[M − H]^−/^[M-C_5_H_9_ − H]^−^	285/330(sh)/360(sh)
C8	4.3	humulone & adhumulone		362 362	361(100)/292(40) 361(100)/292(40)	[M − H]^−/^[M-C_5_H_9_ − H]^−^ [M − H]^−/^[M-C_5_H_9_ − H]^−^	285/330(sh)/360(sh) 285/330(sh)/360(sh)
D7	3.2	postlupulone		386	385(100)	[M − H]^−^	331/271(sh)
D6	1.9	prehumulone & adprehumulone		376 376	375(100)/306(20) 375(100)/306(20)	[M − H]^−/^[M-C_5_H_9_ − H]^−^ [M − H]^−/^[M-C_5_H_9_ − H]^−^	285/330(sh)/360(sh) 285/330(sh)/360(sh)
D5	92.5	colupulone		400	399(100)	[M − H]^−^	332/275
D4	8696.1	colupulone		400	399(100)	[M − H]^−^	332/275
D3	97.4	lupulone		414	413(100)	[M − H]^−^	331/275
D2	88.7	adlupulone		414	413(100)	[M − H]^−^	331/275
E1	0.9	prelupulone & adprelupulone		428 428	427(100) 427(100)	[M − H]^−^ [M − H]^−^	330/275 330/275
**(C) Humulone-enriched extract**							
**Fraction**	**% Mortality**	**In-Well Molar Concentration (** **μM)**	**Principal Component**	**Obs. MW (Da)**	**ESI-NIM (Intensity)**	**Deprotonated Ion and Fragments**	**UV-Vis** **λmax (nm)**
P1-E5	1.9	1.21	cohumulone	348	347(100)/278(40)	[M − H]^−/^[M-C_5_H_9_ − H]^−^	285/330(sh)/360(sh)
P1-E6	0.2	93.8	cohumulone	348	347(100)/278(40)	[M − H]^−/^[M-C_5_H_9_ − H]^−^	285/330(sh)/360(sh)
P1-E7	1.4	31.5	cohumulone	348	347(100)/278(40)	[M − H]^−/^[M-C_5_H_9_ − H]^−^	285/330(sh)/360(sh)
P1-F5	57	92.0	humulone	362	361(100)/292(40)	[M − H]^−/^[M-C_5_H_9_ − H]^−^	285/330(sh)/360(sh)
P1-F4	42	142.0	humulone	362	361(100)/292(40)	[M − H]^−/^[M-C_5_H_9_ − H]^−^	285/330(sh)/360(sh)
P1-F3	2.7	57.2	humulone	362	361(100)/292(40)	[M − H]^−/^[M-C_5_H_9_ − H]^−^	285/330(sh)/360(sh)
P1-F2	2.2	16.6	adhumulone	362	361(100)/292(40)	[M − H]^−/^[M-C_5_H_9_ − H]^−^	285/330(sh)/360(sh)
P1-F1	1.4	14.1	adhumulone	362	361(100)/292(40)	[M − H]^−/^[M-C_5_H_9_ − H]^−^	285/330(sh)/360(sh)
P2-A1	0.6	2.3	adhumulone	362	361(100)/292(40)	[M − H]^−/^[M-C_5_H_9_ − H]^−^	285/330(sh)/360(sh)
P2-A7	1.4	n/c	postlupulone	386	385(100)	[M − H]^−^	331/271
P2-B2	40.9	n/c	colupulone	400	399(100)	[M − H]^−^	332/275
**(D) Lupulone-enriched extract**							
**Fraction**	**% Mortality**	**In-Well Molar Concentration (** **μM)**	**Principal Component**	**Obs. MW (Da)**	**ESI-NIM (Intensity)**	**Deprotonated Ion and Fragments**	**UV-Vis** **λmax (nm)**
P1-F6	33	30.5	colupulone	400	399(100)	[M − H]^−^	332/275
P1-F5	33	31.0	colupulone	400	399(100)	[M − H]^−^	332/275
P1-F4	10	8.24	colupulone	400	399(100)	[M − H]^−^	332/275
P2-A3	100	29.0	lupulone	414	413(100)	[M − H]^−^	331/275
P2-A4	64	11.2	lupulone	414	413(100)	[M − H]^−^	331/275
P2-A5	66	10.3	adlupulone	414	413(100)	[M − H]^−^	331/275
P2-A6	9.1	5.31	adlupulone	414	413(100)	[M − H]^−^	331/275

Obs. MW = observed molecular weight; ESI = electrospray ionization; NIM = negative ion mode. * tentative identification for trace components.

## Supplementary Materials

The following are available online at https://www.mdpi.com/1420-3049/25/16/3677/s1.
