# Supplementary material for: Ultra-Micro-Scale-Fractionation (UMSF) as a Powerful Tool for Bioactive Molecules Discovery
Source: Molecules. 2020 Aug 12;25(16):3677. doi: 10.3390/molecules25163677 (PMC7464926; doi:10.3390/molecules25163677)

Supplementary Figure 1A: 1 minute retention time window components
Hops F6 (5-6 minutes)

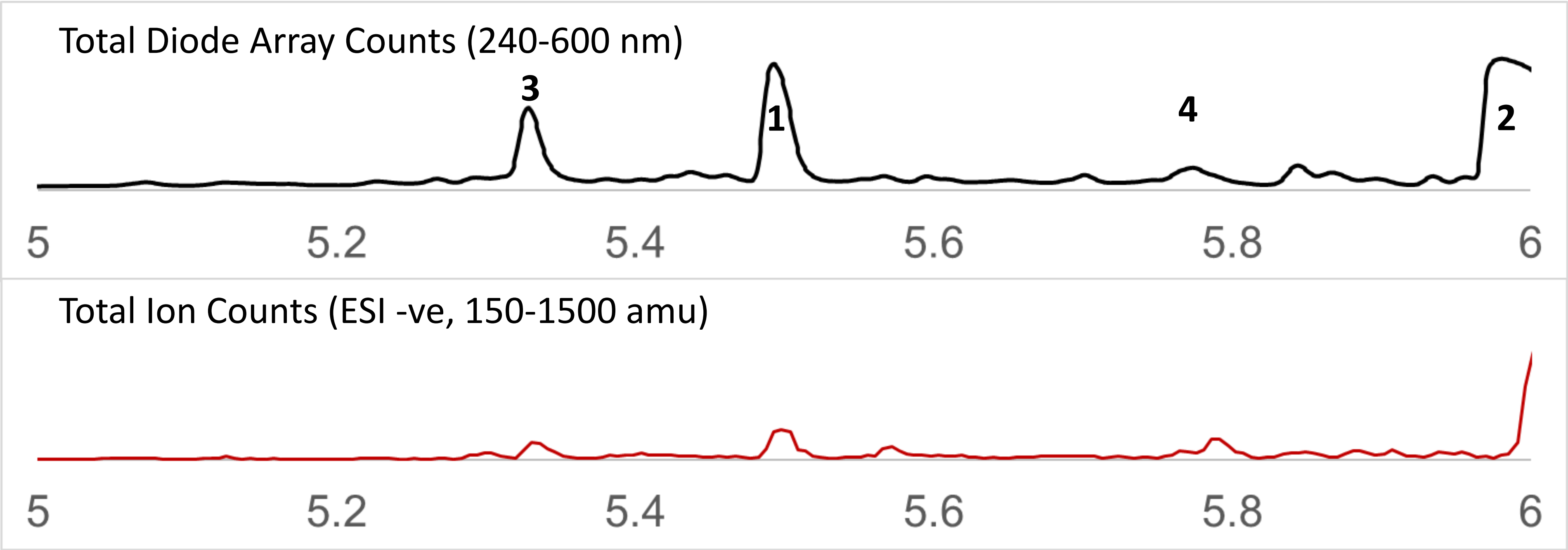

Supplementary Figure 1A: 1 minute retention time window components
Hops F6 (5-6 minutes) Major Components

1 – xanthohumol
RT: 5.48 – 5.52 minutes

2 – cohumulone
RT: 5.95-6.03 minutes

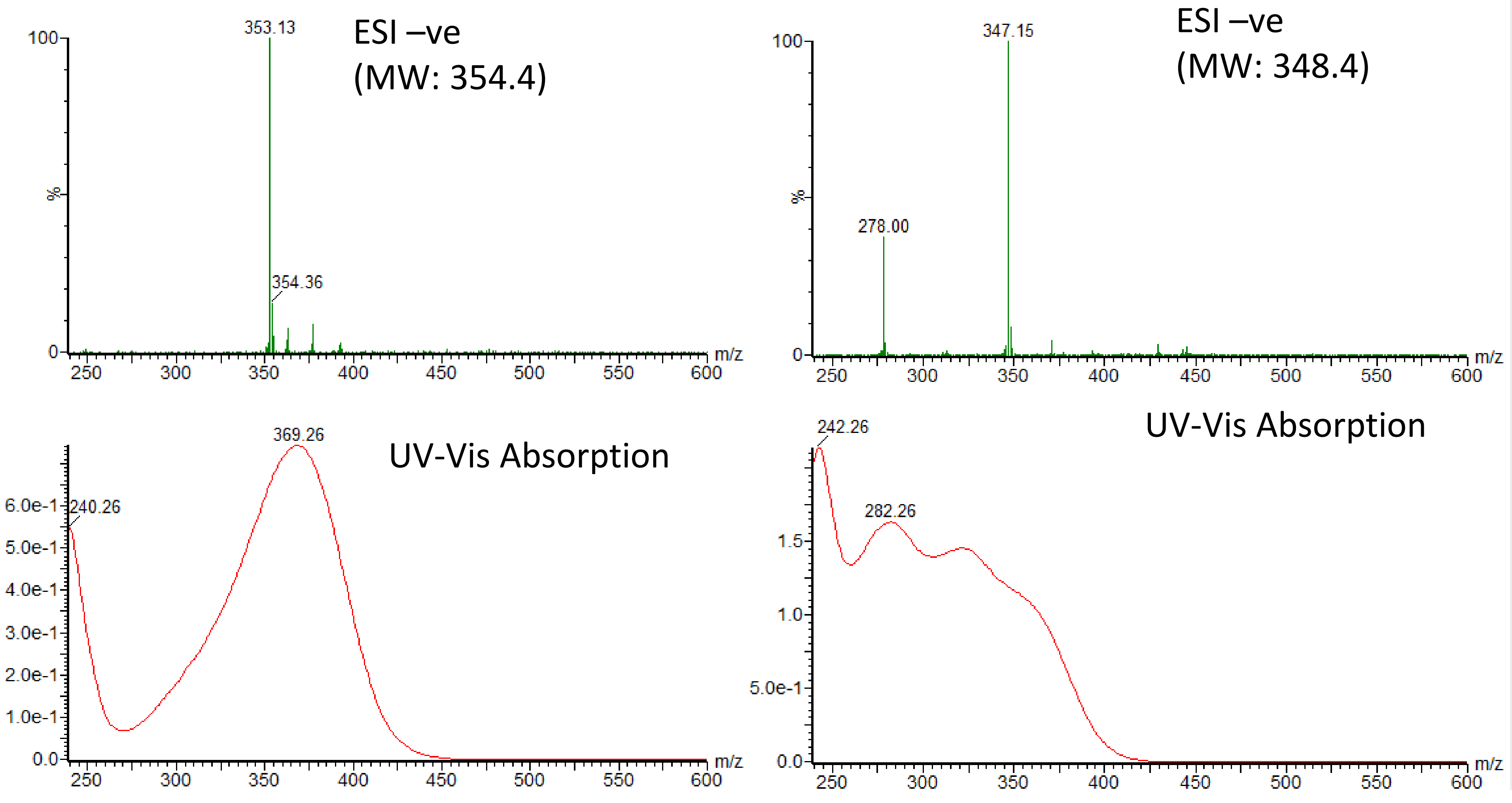

Supplementary Figure 1A: 1 minute retention time window components
Hops F6 (5-6 minutes) Minor Components

3 – desmethylxanthohumol
RT: 5.315 – 5.35 min

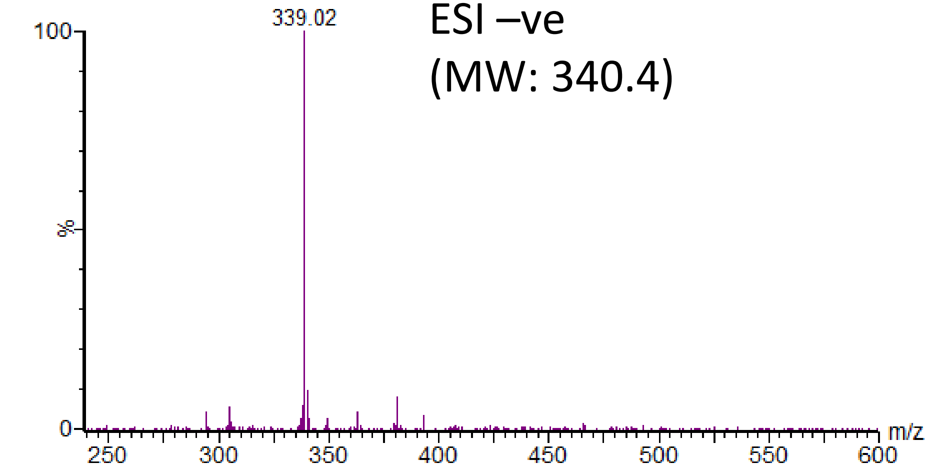

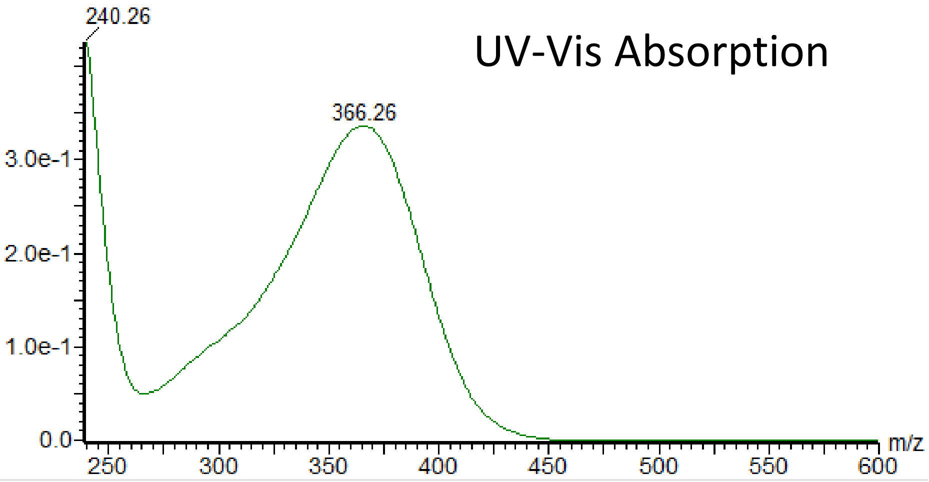

4 – post lupulone
RT: 5.83 – 5.87 min

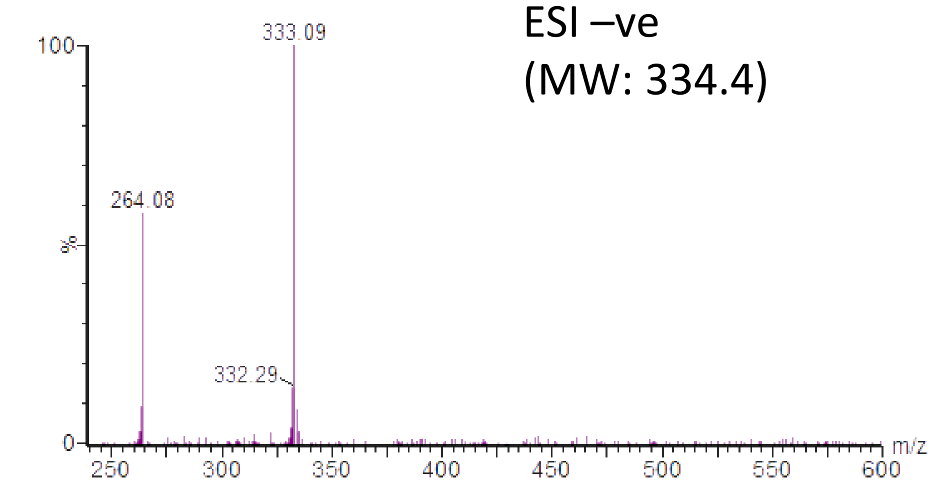

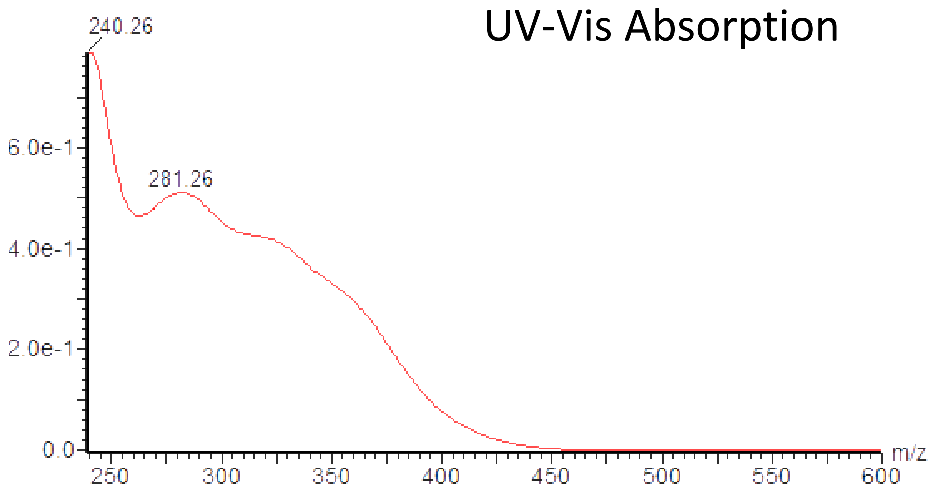

Supplementary Figure 1B: 1 minute retention time window components
Hops F7 (6-7 minutes)

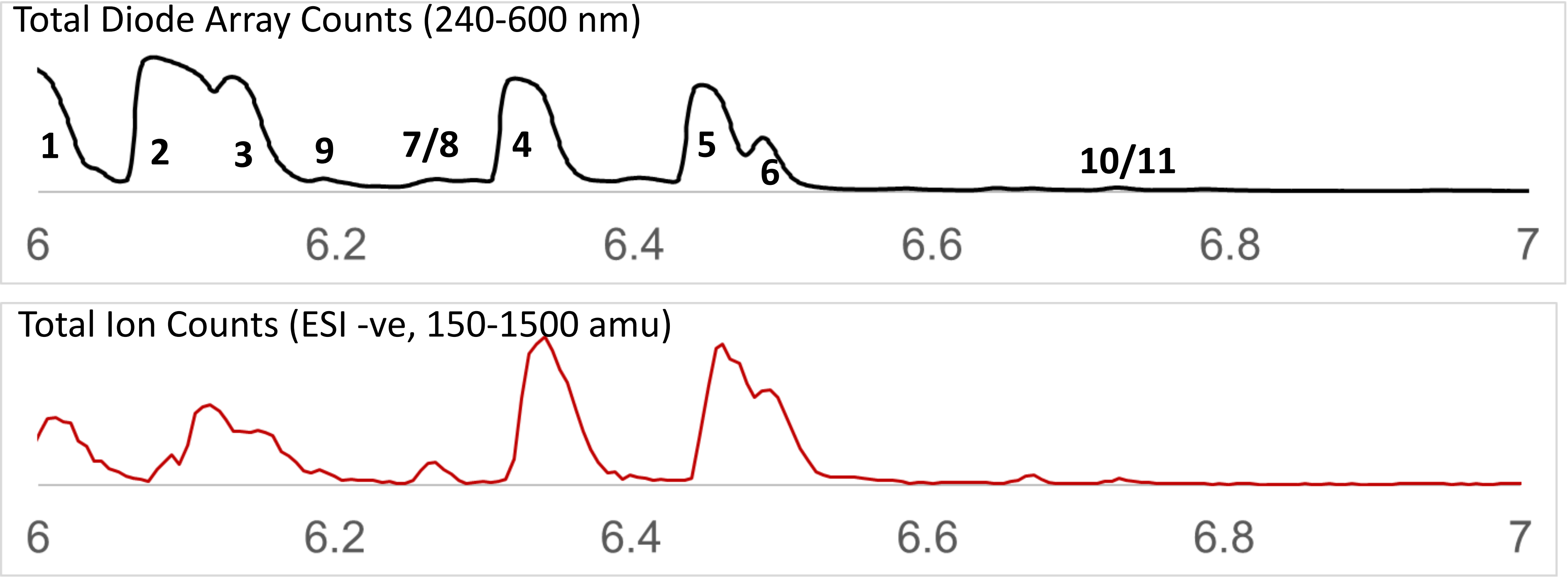

Supplementary Figure 1B: 1 minute retention time window components
Hops F7 (6-7 minutes) Major Components

1 – cohumulone
RT: 5.95-6.03 minutes

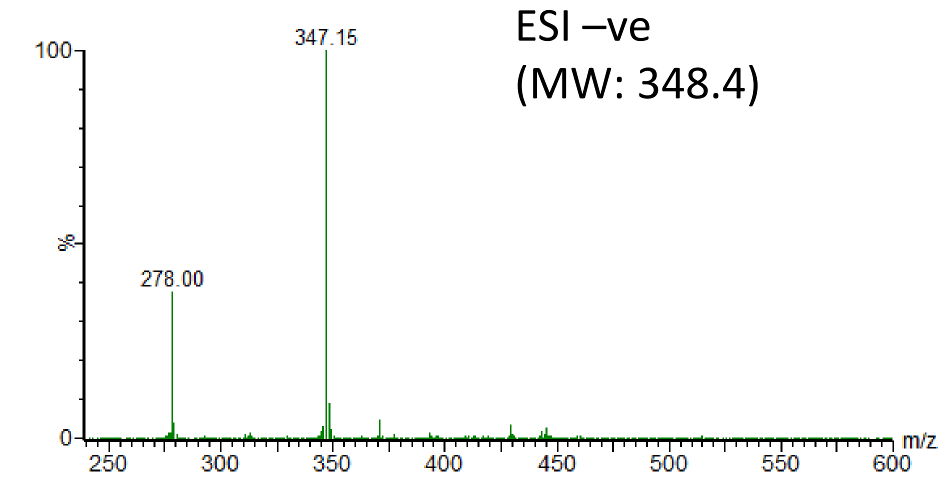

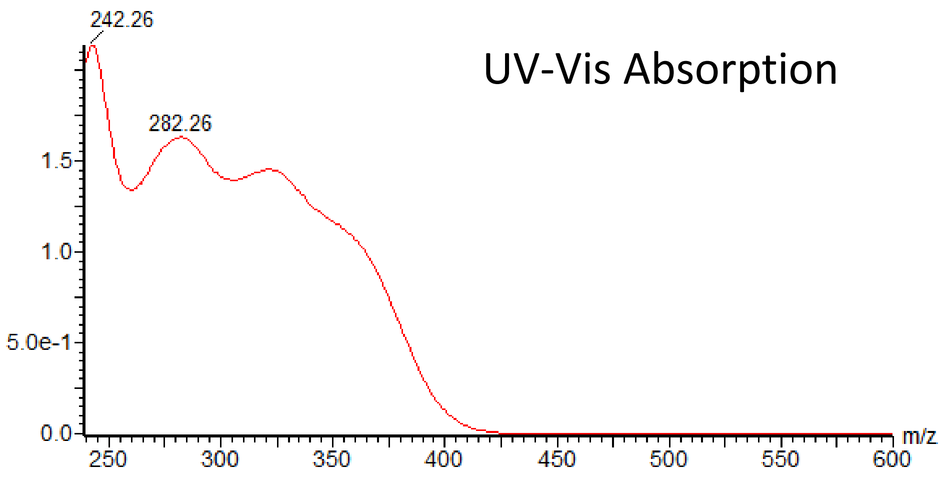

2 – humulone
RT: 6.06-6.13 minutes

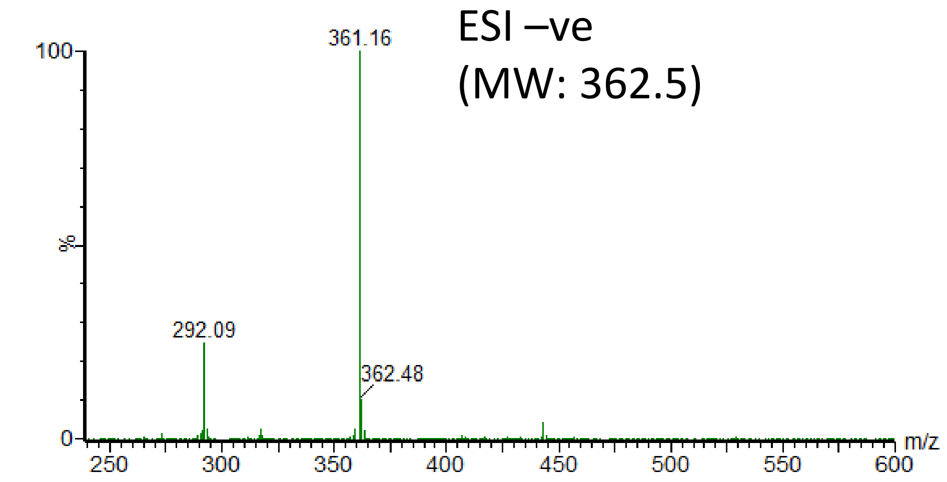

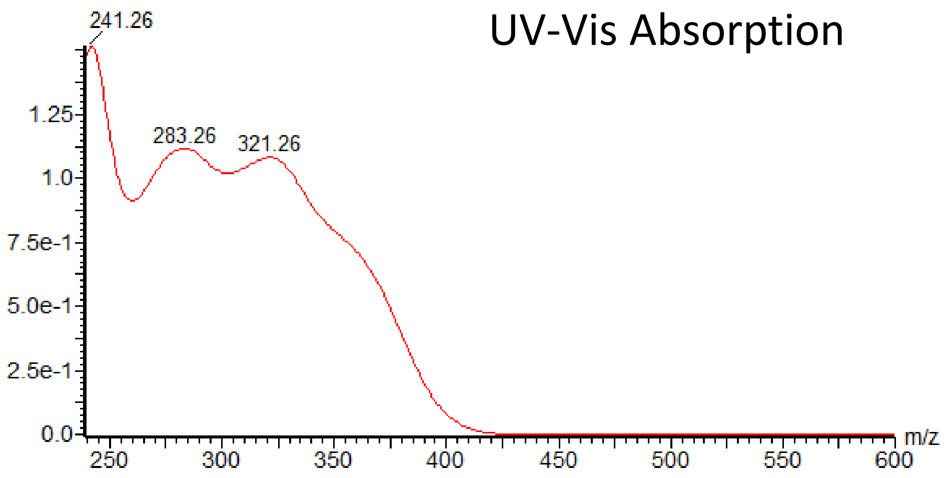

Supplementary Figure 1B: 1 minute retention time window components
Hops F7 (6-7 minutes) Major Components

3 – adhumulone
RT: 6.11-6.17 minutes

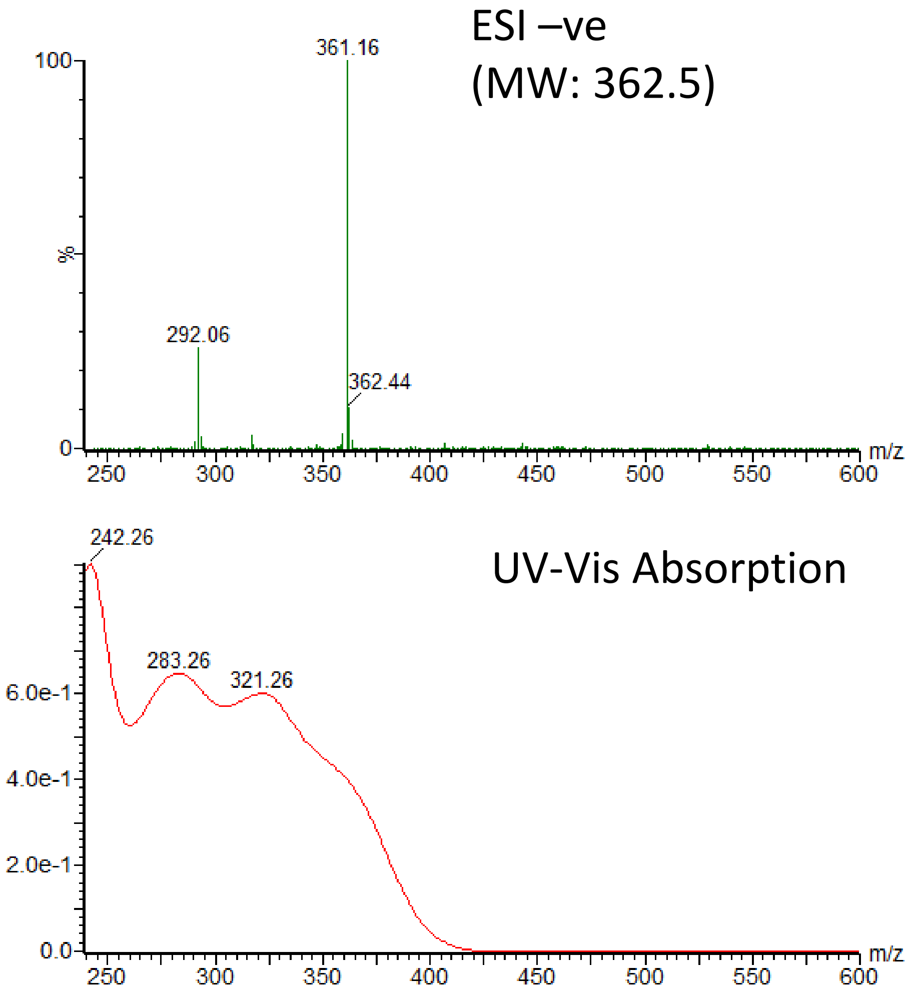

4 – colupulone
RT: 6.31-6.36 minutes

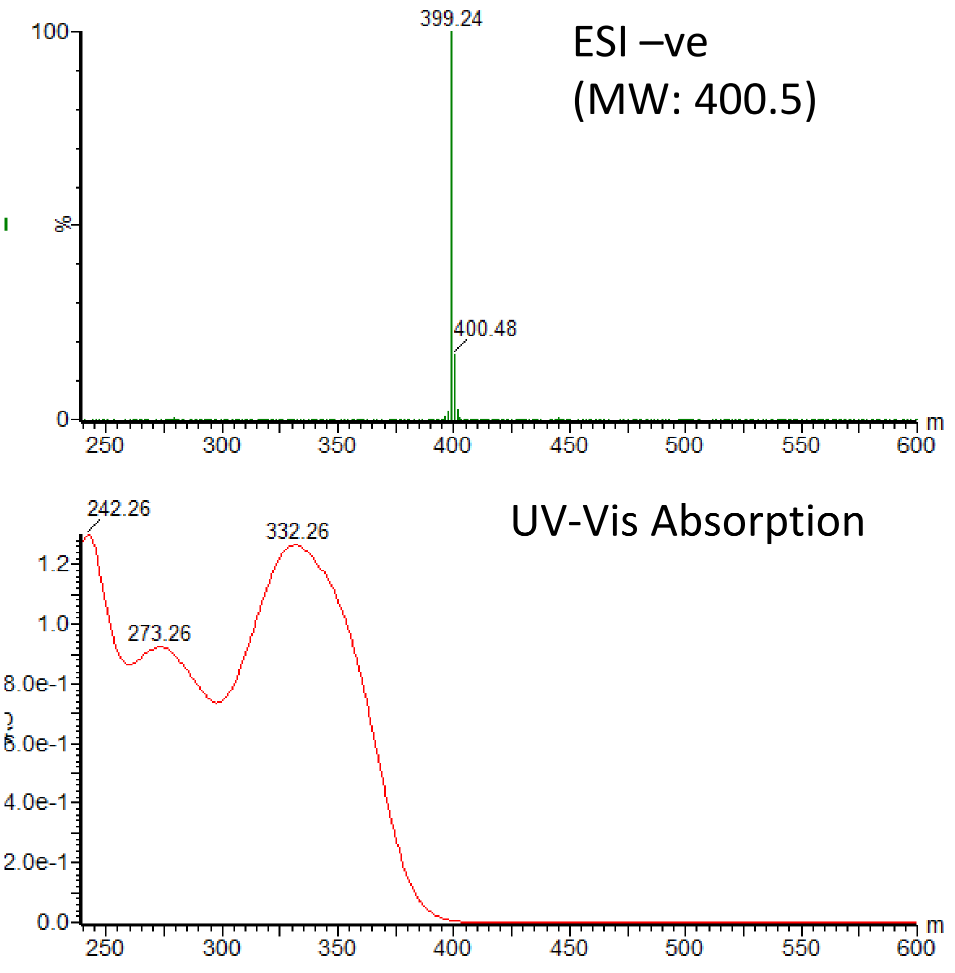

Supplementary Figure 1B: 1 minute retention time window components
Hops F7 (6-7 minutes) Major Components

5 – lupulone
RT: 6.43-6.47 minutes

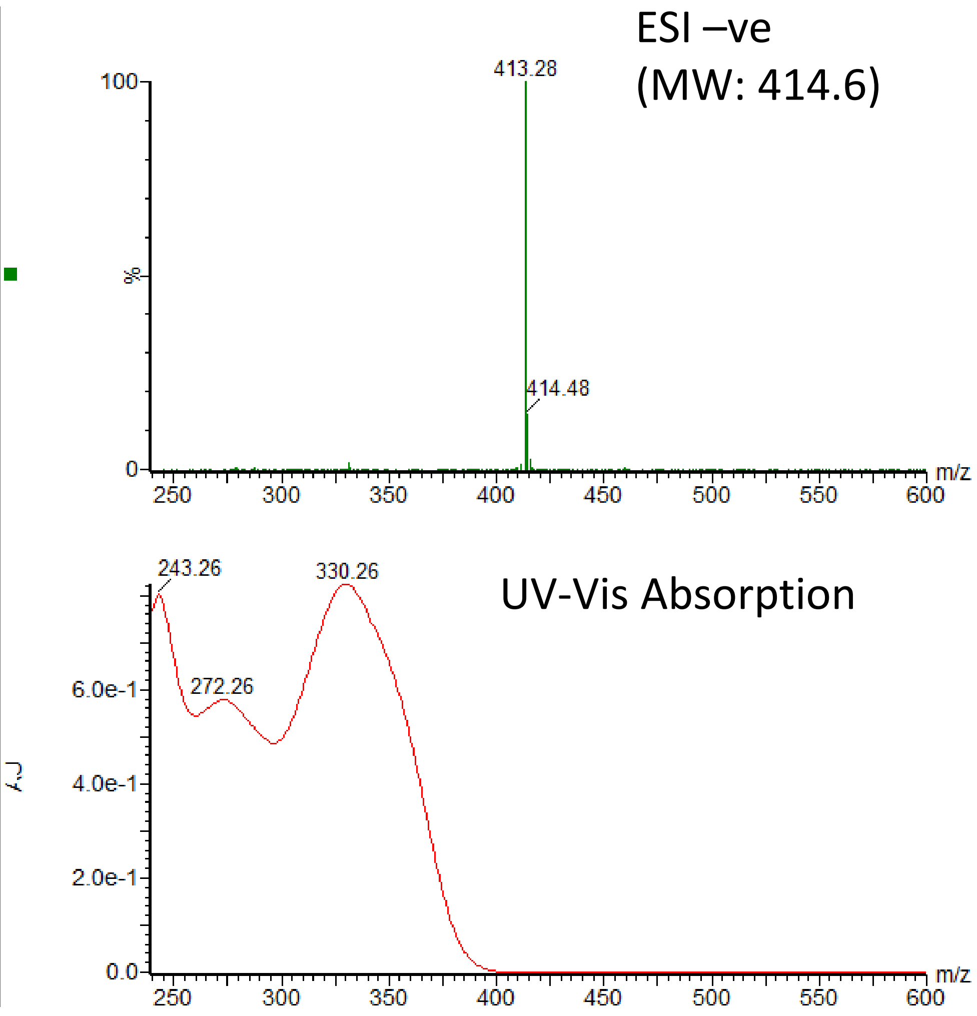

6 – adlupulone
RT: 6.47-6.52 minutes

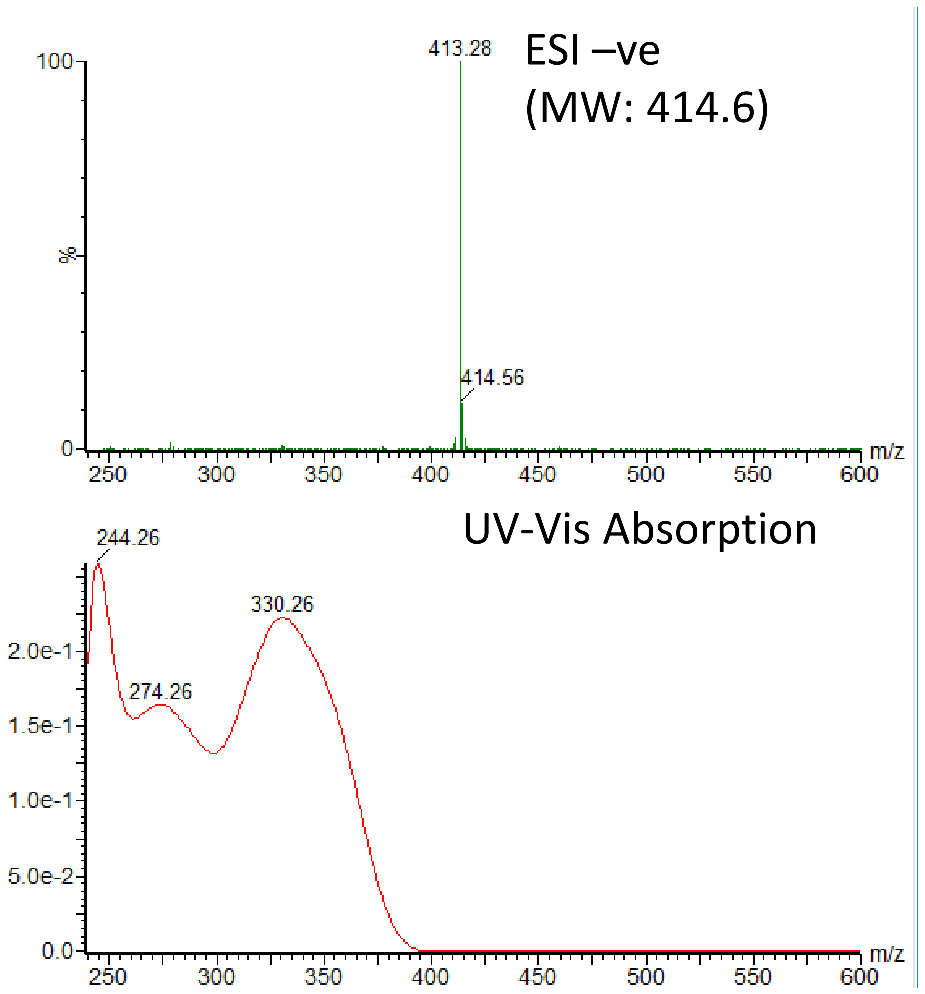

Supplementary Figure 1B: 1 minute retention time window components
Hops F7 (6-7 minutes) Minor Components

7/8 – prehumulone & adprehumulone
RT: 6.28-6.32 minutes

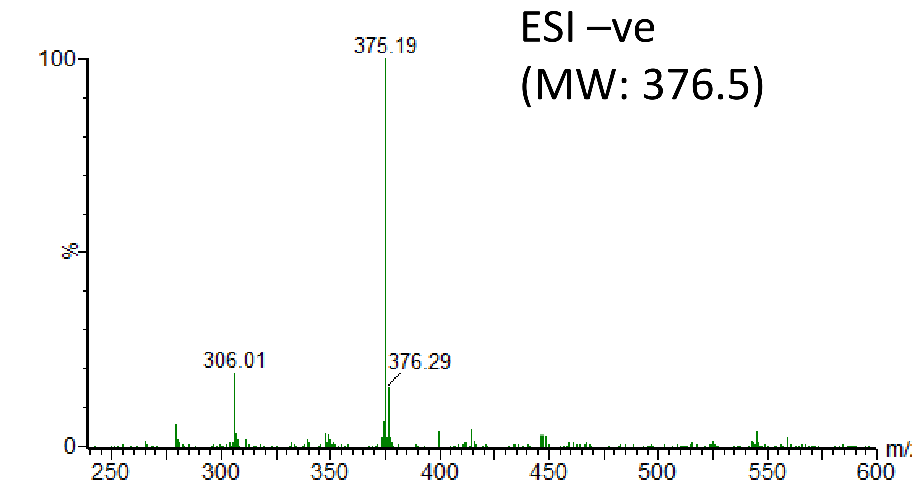

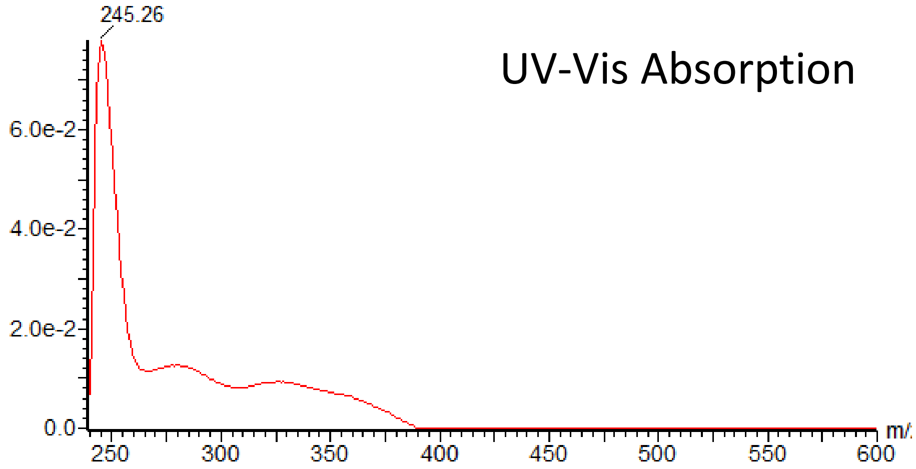

9 – postlupulone
RT: 6.17-6.22 minutes

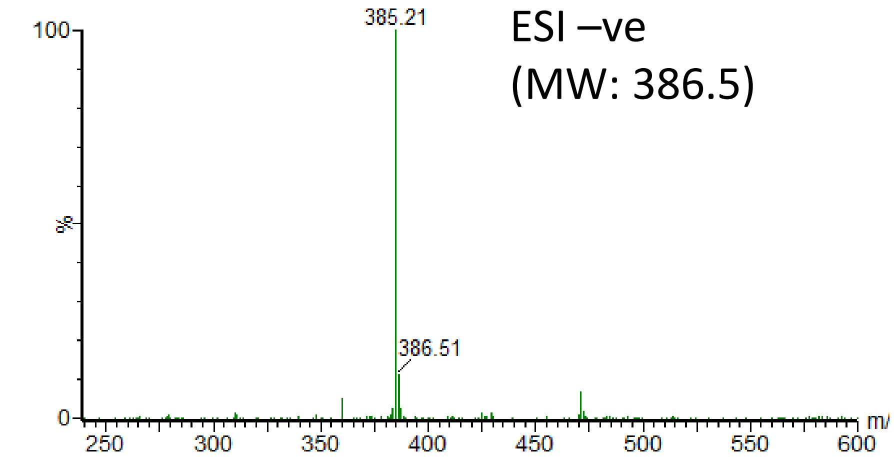

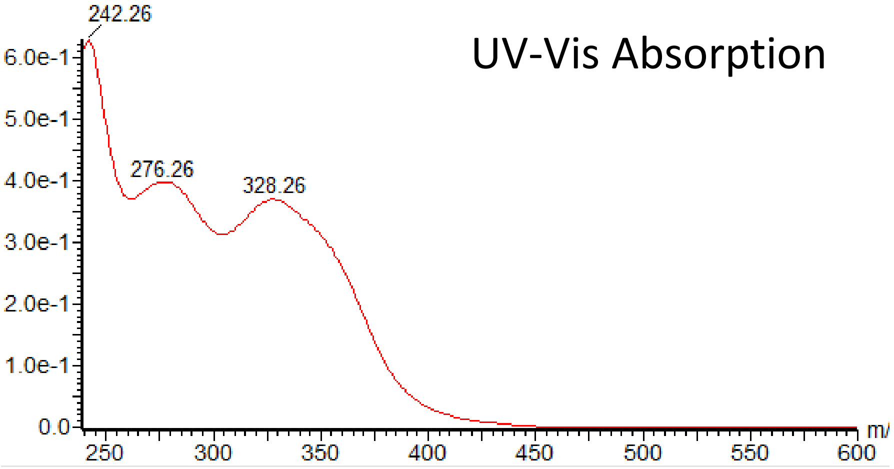

Supplementary Figure 1B: 1 minute retention time window components
Hops F7 (6-7 minutes) Minor Components

10/11 – prelupulone & adprelupulone
RT: 6.62-6.68 minutes

ESI –ve
(MW: 428.6)

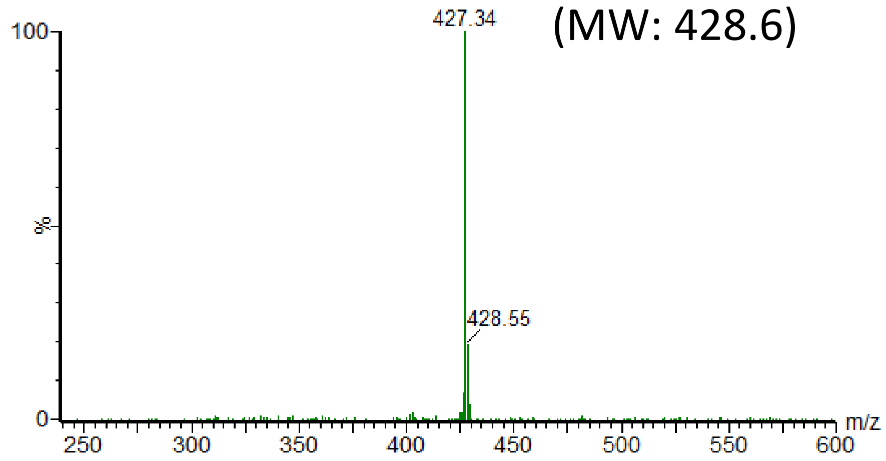

UV-Vis Absorption

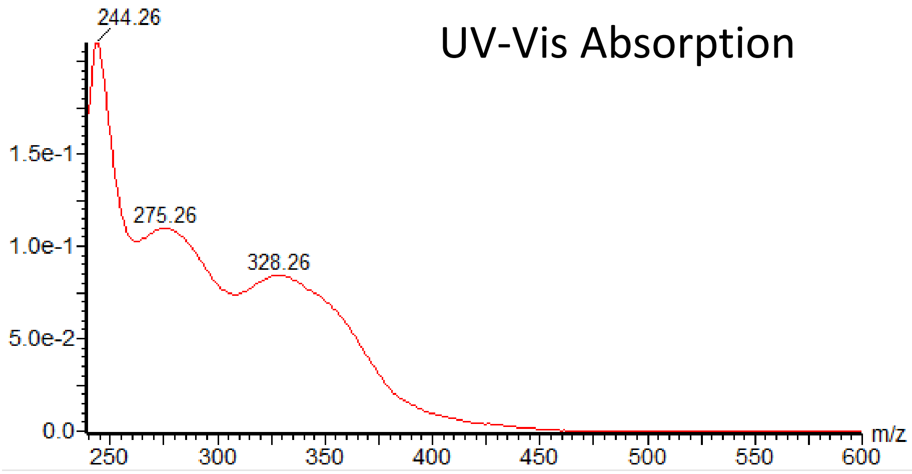

Supplementary Figure 2A: 10 second retention time window components

desmethylxanthohumol
RT: 1.20-1.66 minutes

xanthohumol
RT: 1.80-2.15 minutes

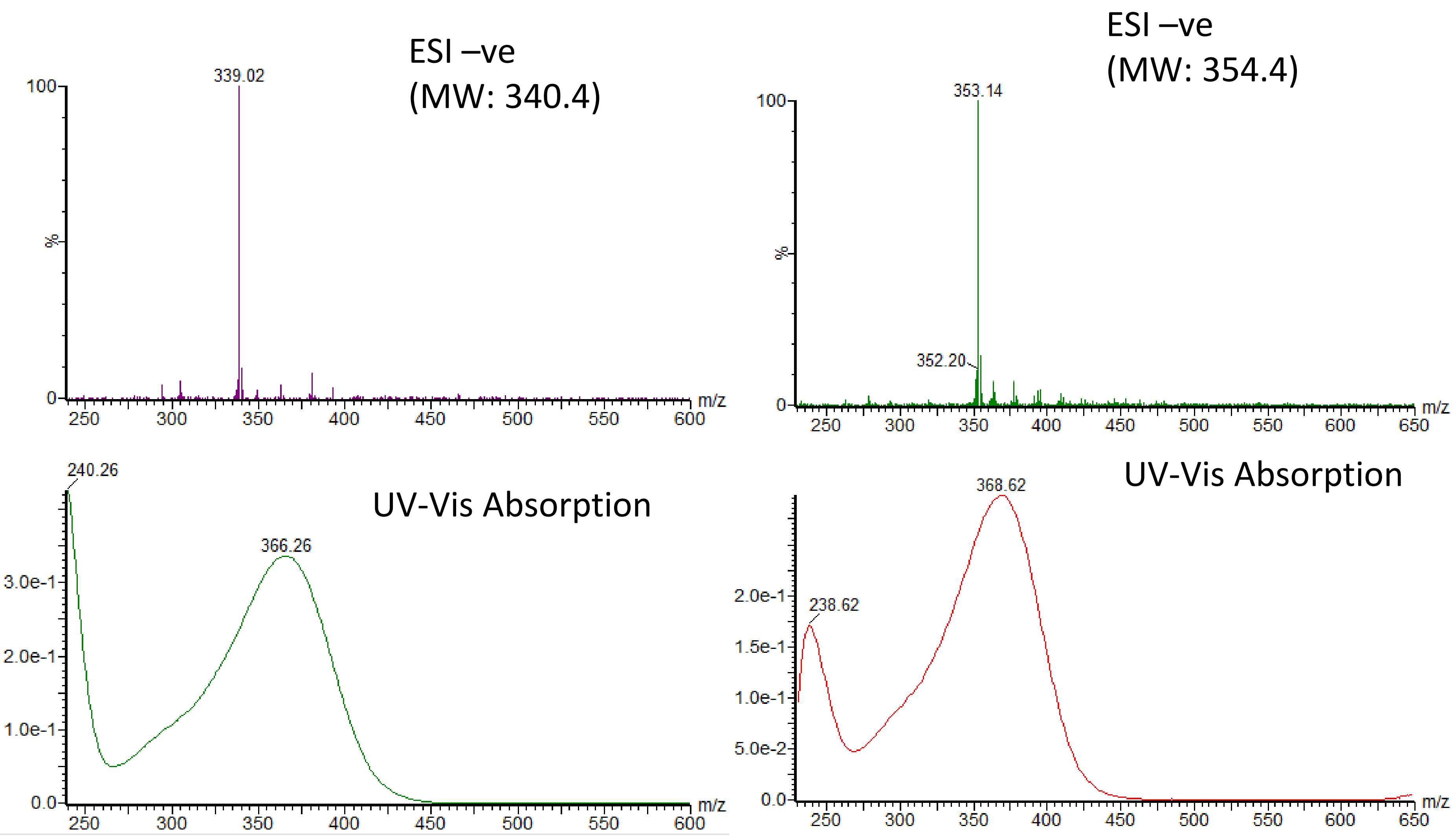

Supplementary Figure 2A: 10 second retention time window components

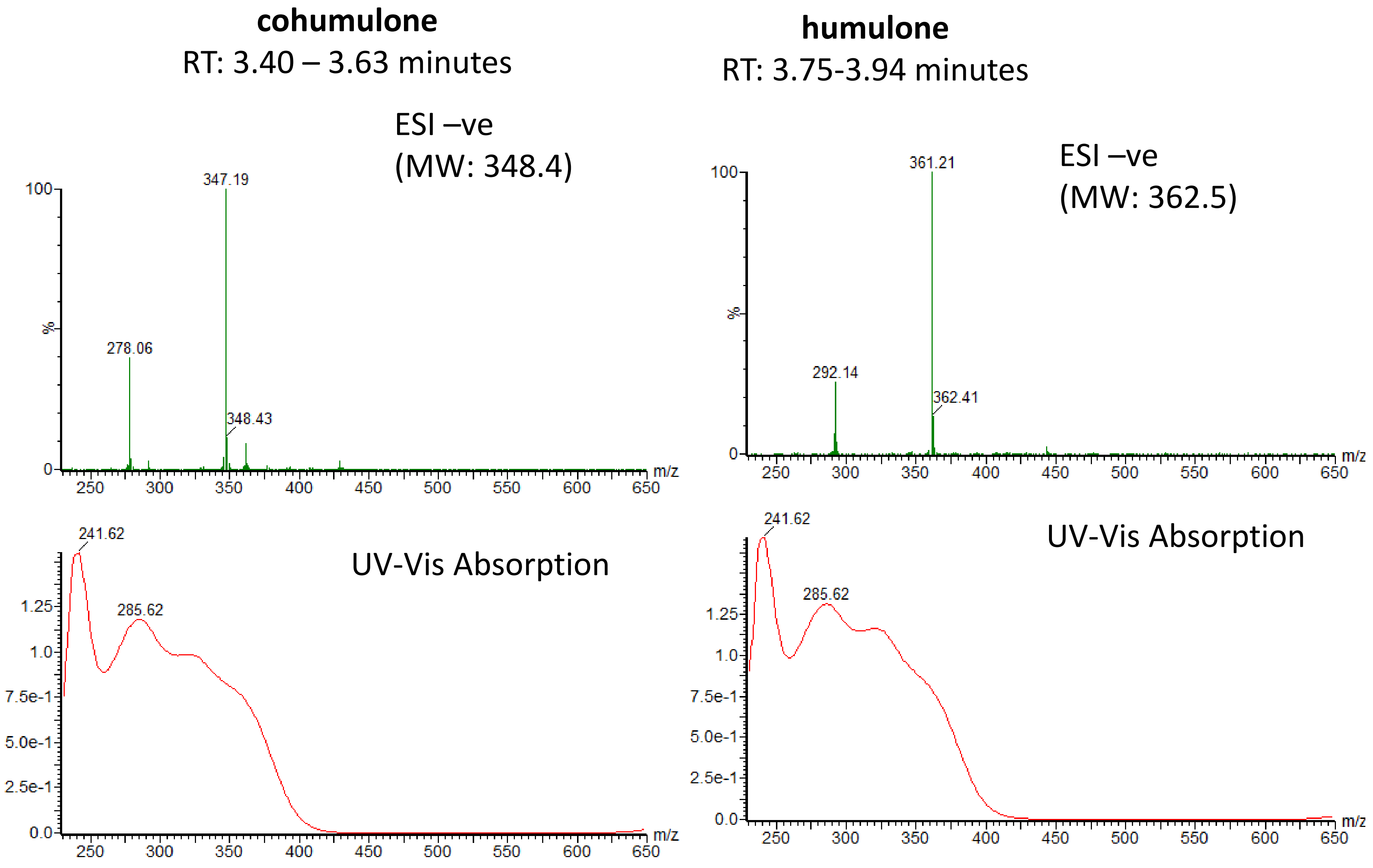

Supplementary Figure 2A: 10 second retention time window components

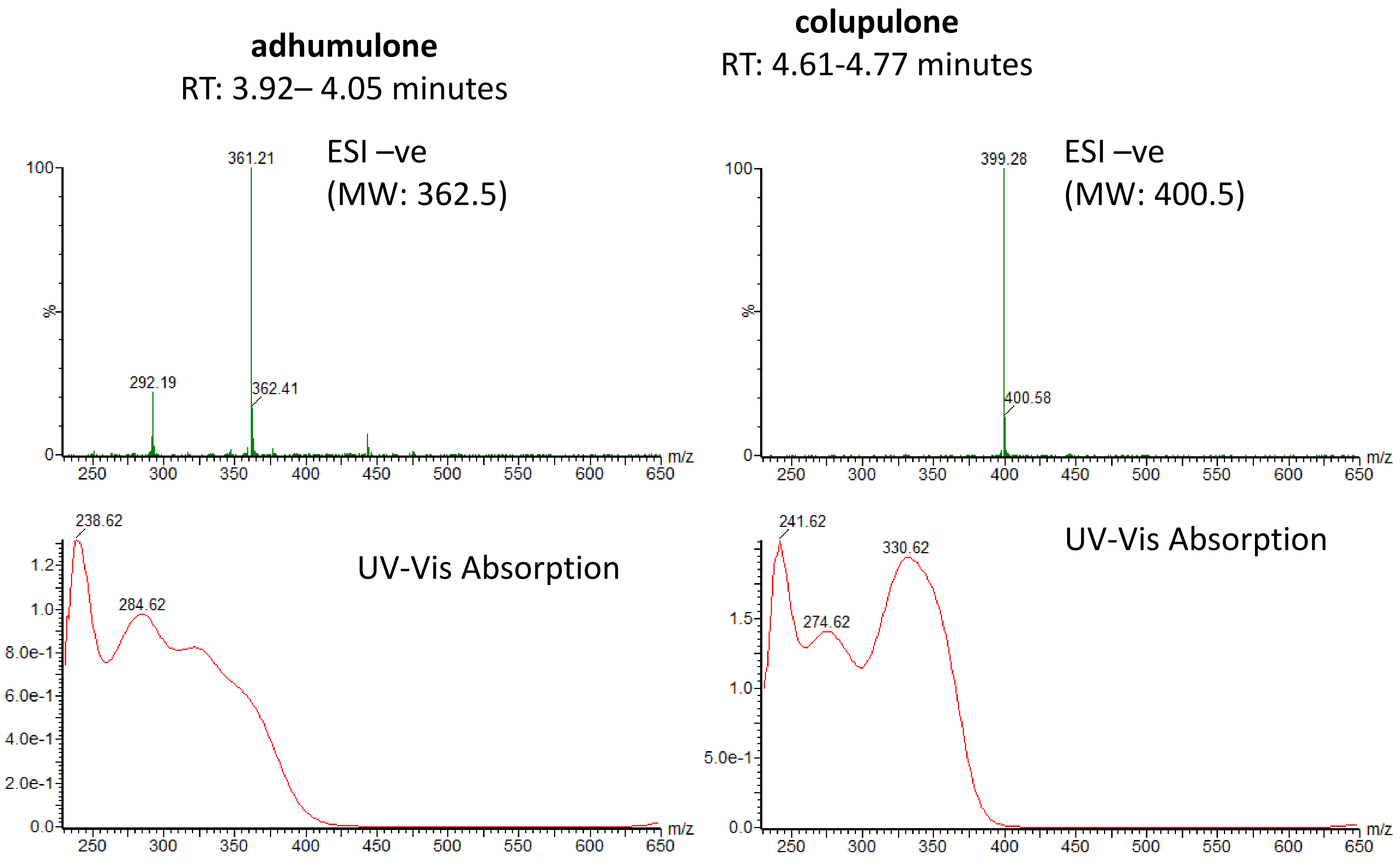

Supplementary Figure 2A: 10 second retention time window components

lupulone

RT: 4.90-5.09 minutes

ESI –ve

(MW: 414.6)

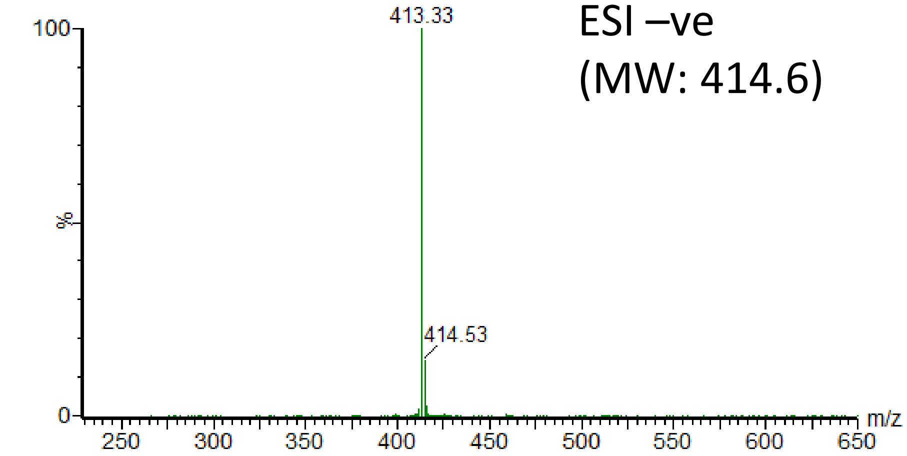

UV-Vis Absorption

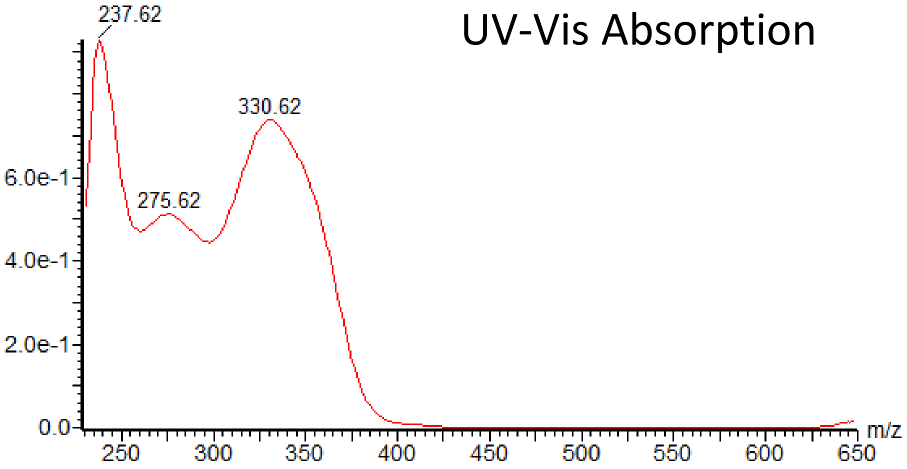

adlupulone

RT: 5.07-5.15 minutes

ESI –ve

(MW: 414.6)

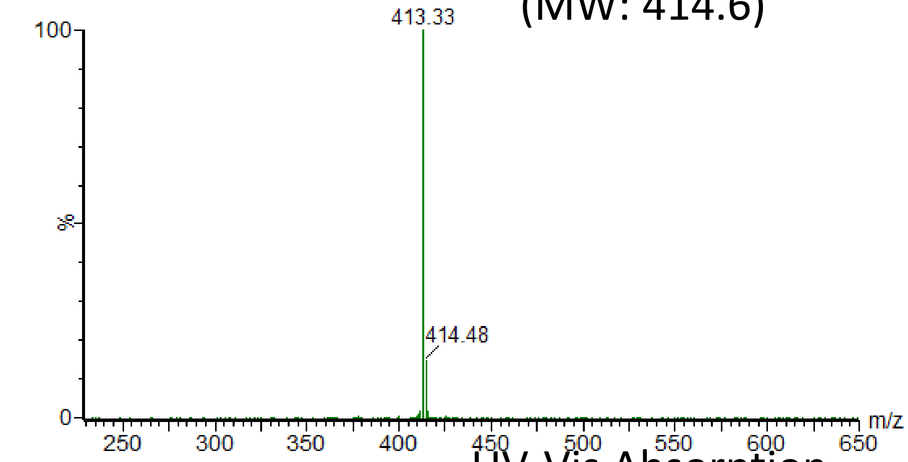

UV-Vis Absorption

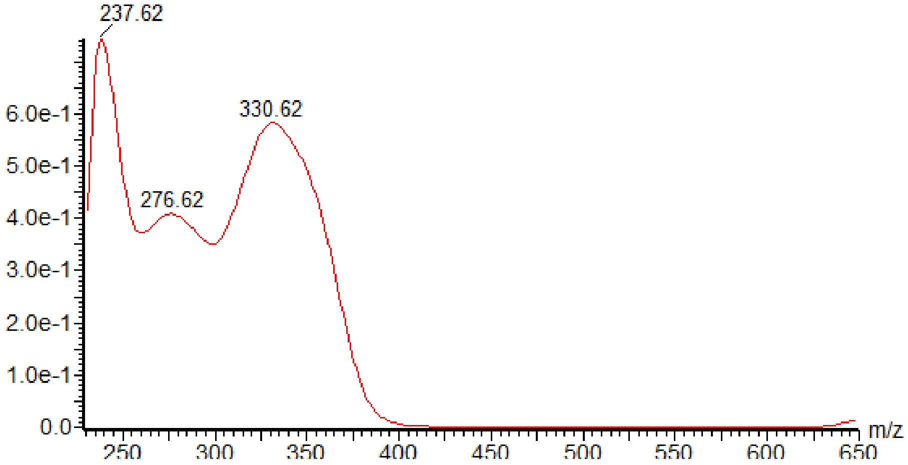

Supplementary Figure 2A: 10 second retention time window components

postlupulone

RT: 4.27-4.35 minutes

ESI –ve
(MW: 386.5)

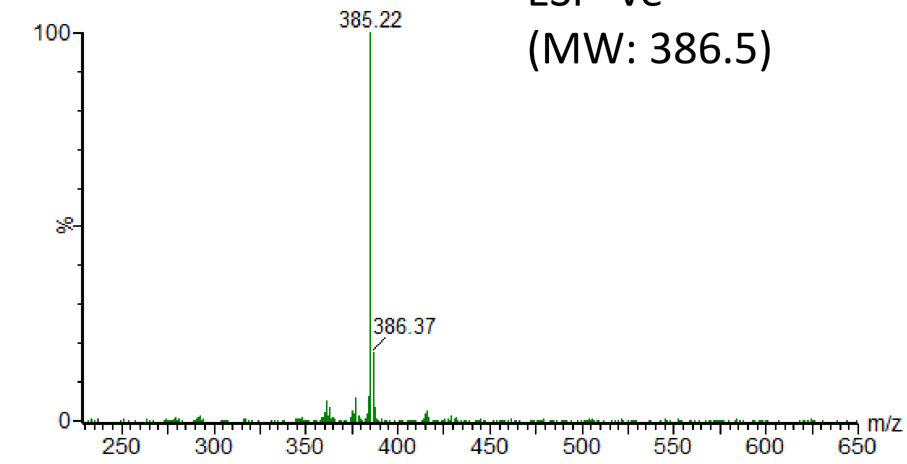

UV-Vis Absorption

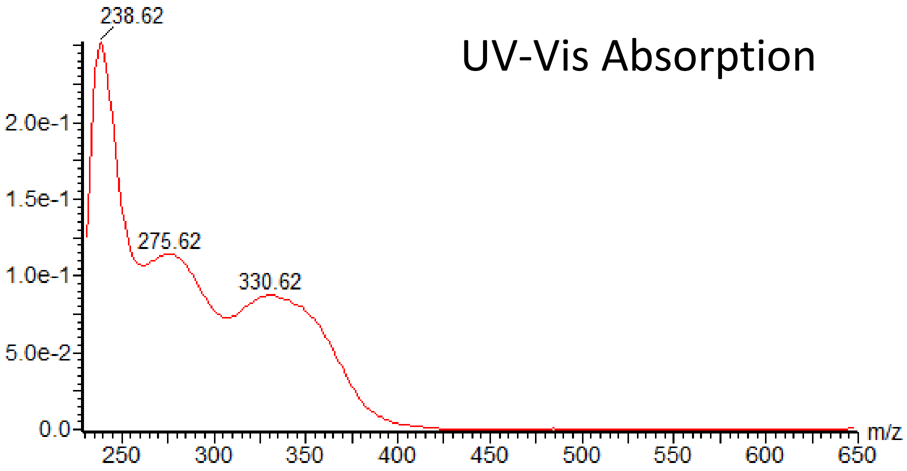

prehumulone & adprehumulone

RT: 4.43-4.58 minutes

ESI –ve
(MW: 376.5)

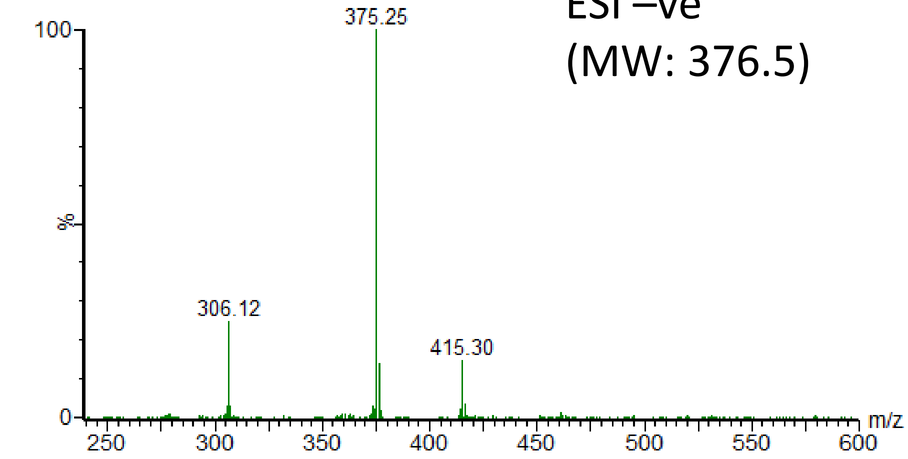

UV-Vis Absorption

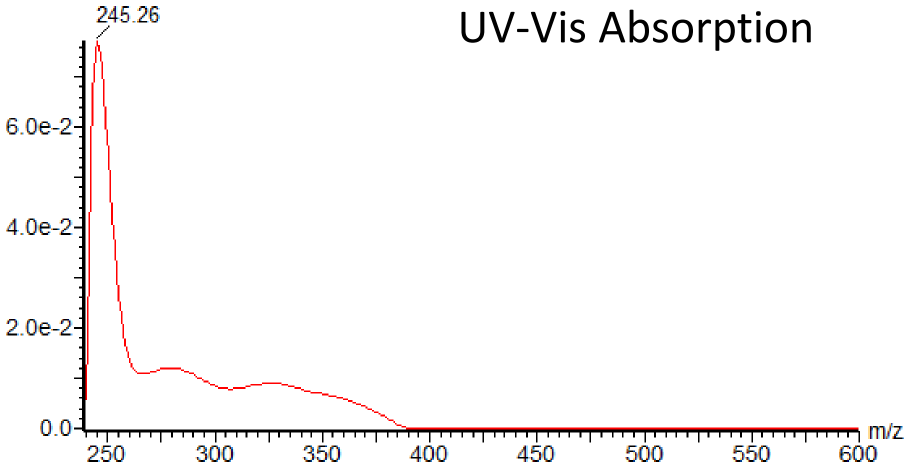

Supplementary Figure 1C: 5 second retention time window components alpha acid enriched extract

cohumulone

RT: 3.01- 3.30 minutes (wells E6-E8 Plate 1)

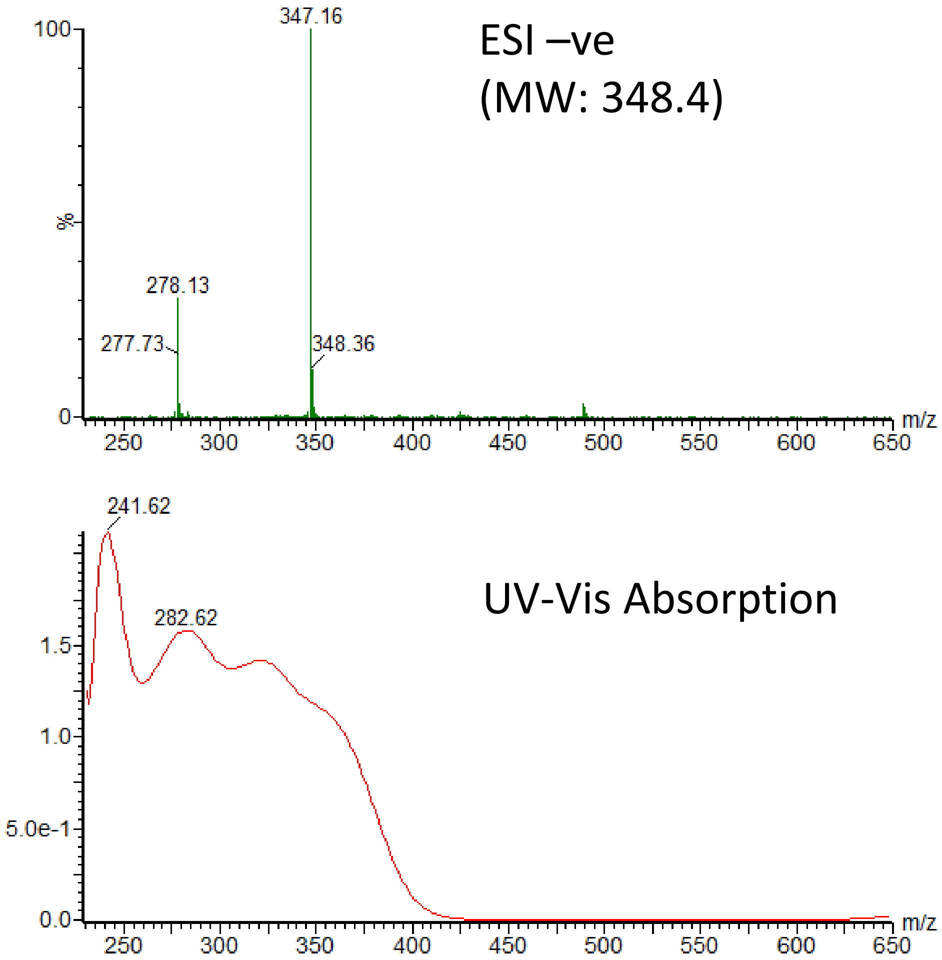

humulone

RT: 3.58- 3.82 minutes (wells F5-F3 Plate 1)

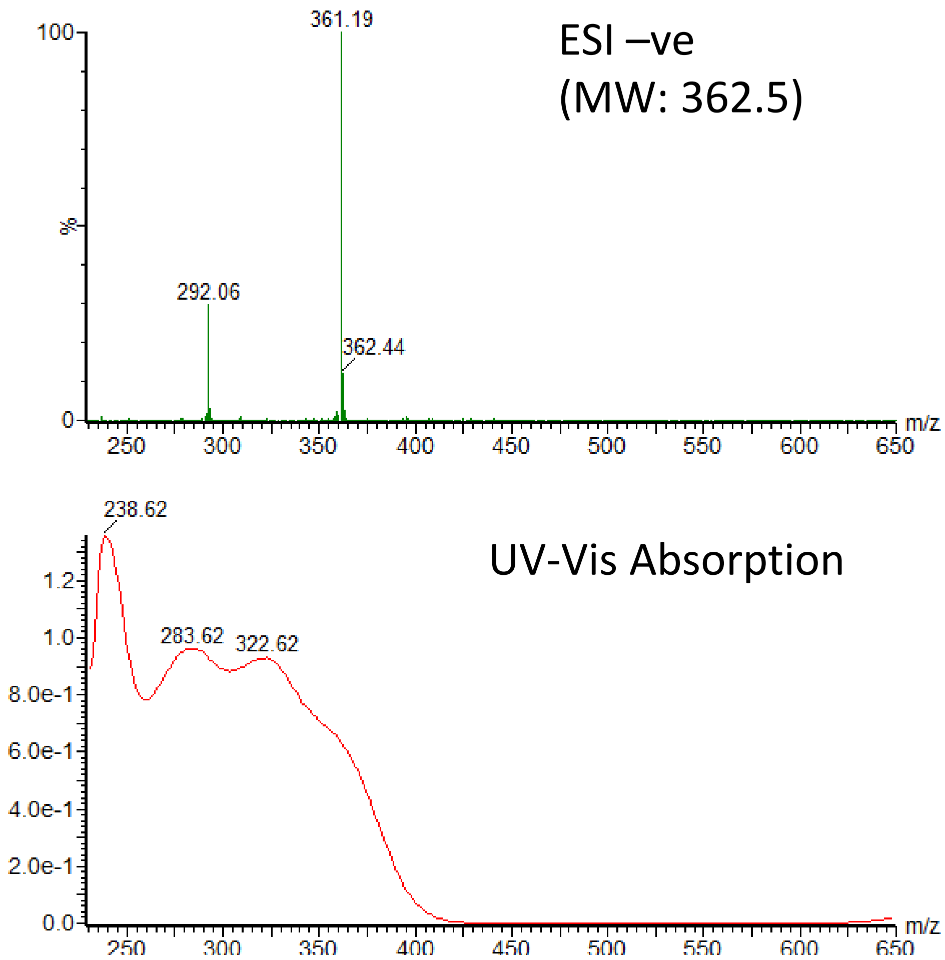

Supplementary Figure 1C: 5 second retention time window components alpha acid enriched extract

adhumulone

RT: 3.81- 4.05 minutes (wells F2-F1 Plate 1)

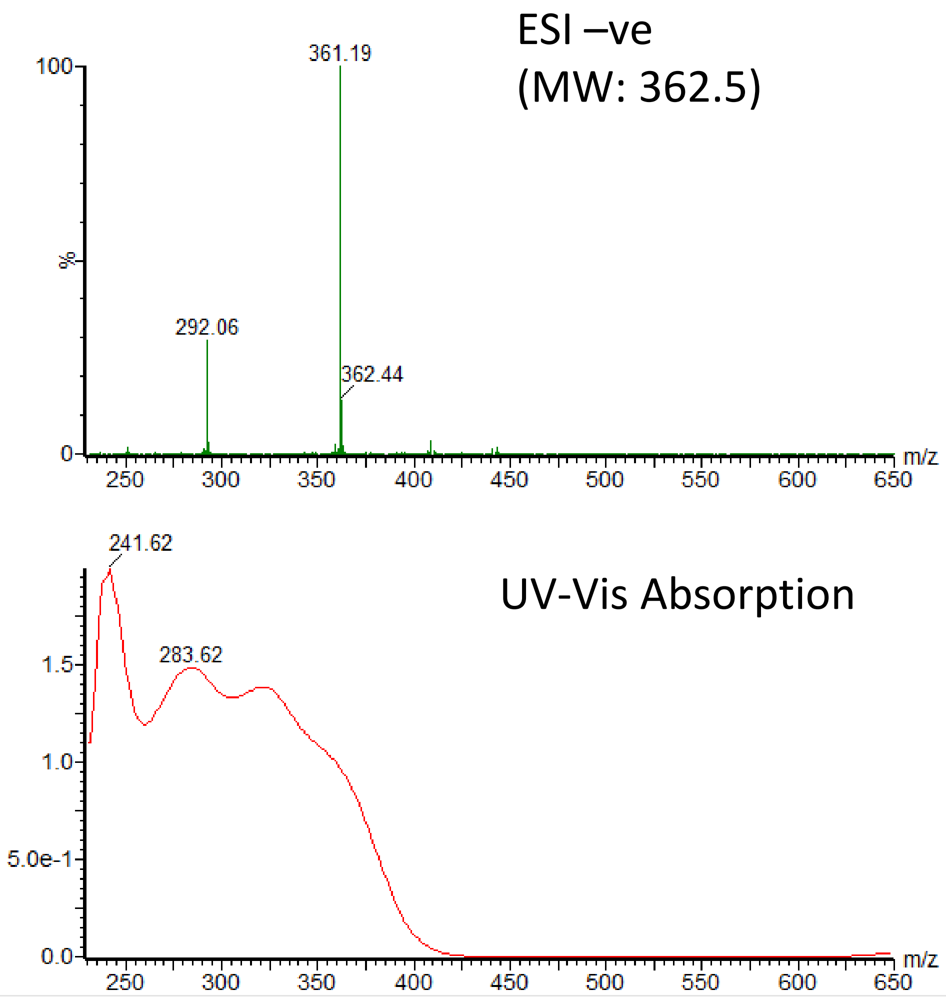

postlupulone

RT: 4.45- 4.52 minutes (well A7 Plate 2)

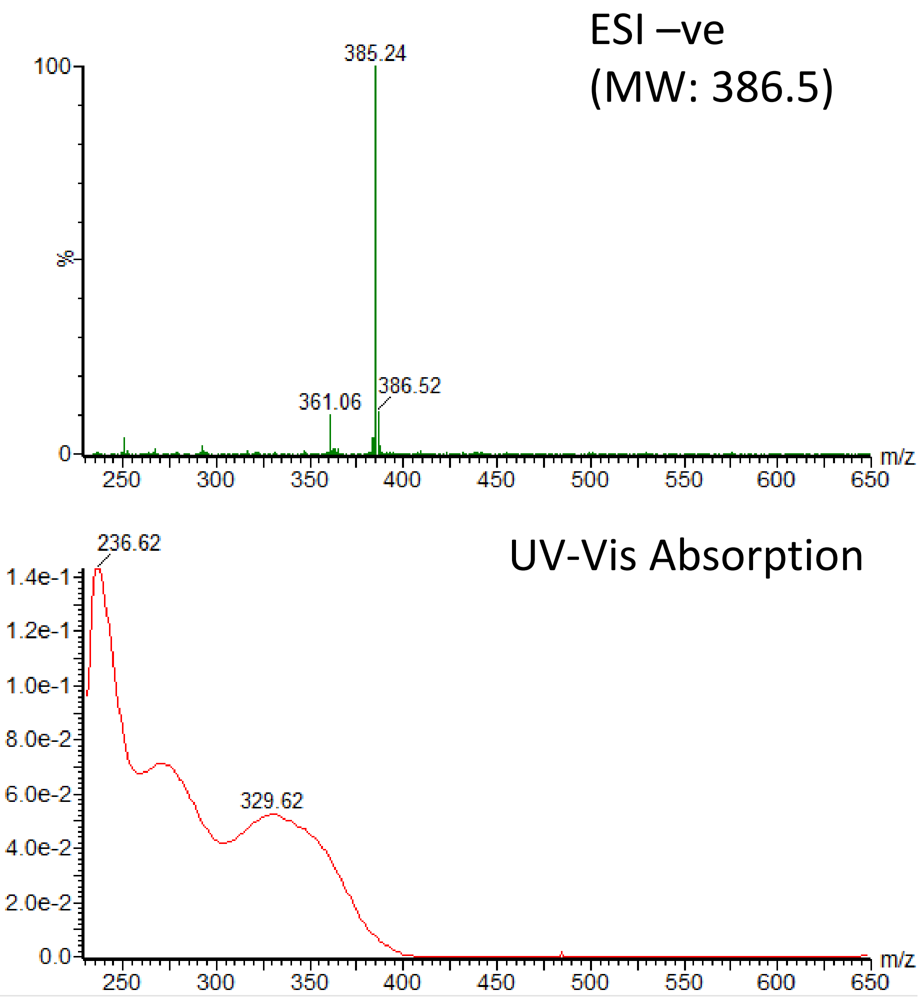

Supplementary Figure 1C: 5 second retention time window components alpha acid enriched extract

colupulone

RT: 5.12- 5.22 minutes (well B2 Plate 2)

ESI –ve

(MW: 400.5)

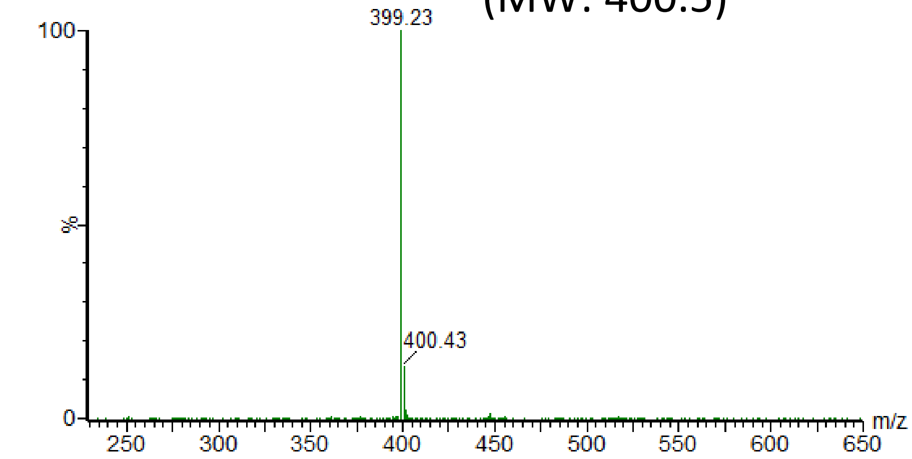

UV-Vis Absorption

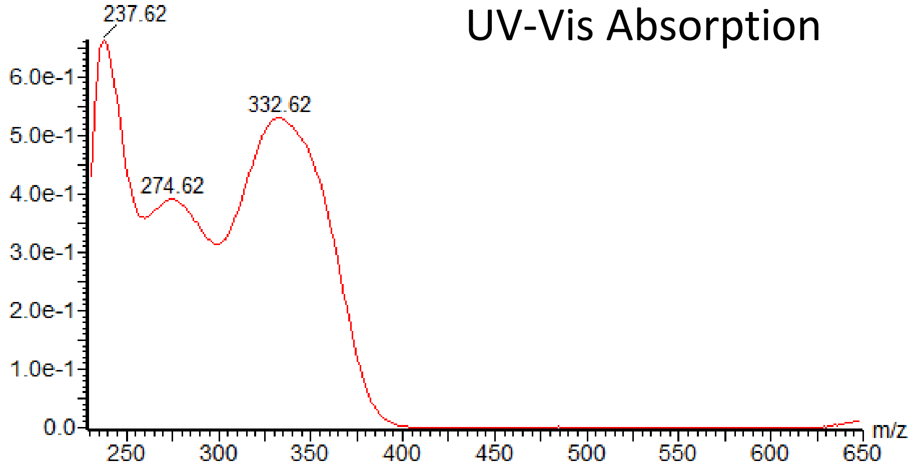

Supplementary Figure 1D: 5 second retention time window components beta acid enriched extract

colupulone

RT: 3.52 – 3.79 minutes (wells F6-F4 Plate 1)

ESI –ve
(MW: 400.5)

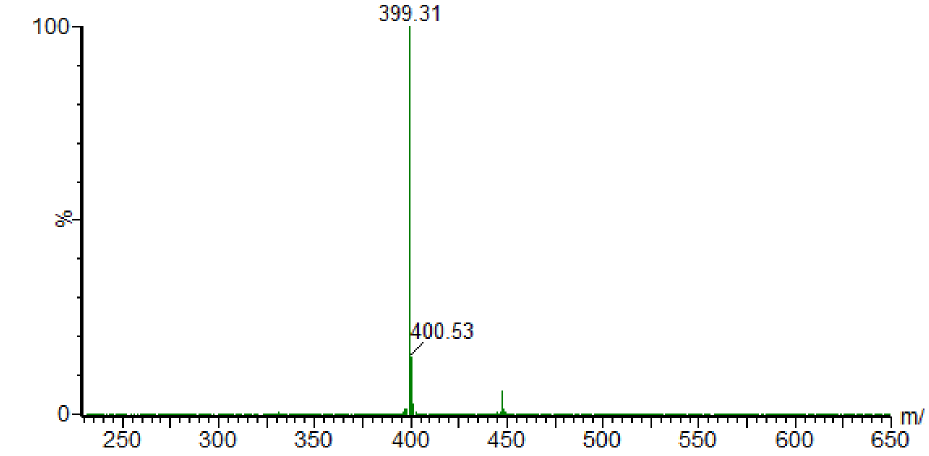

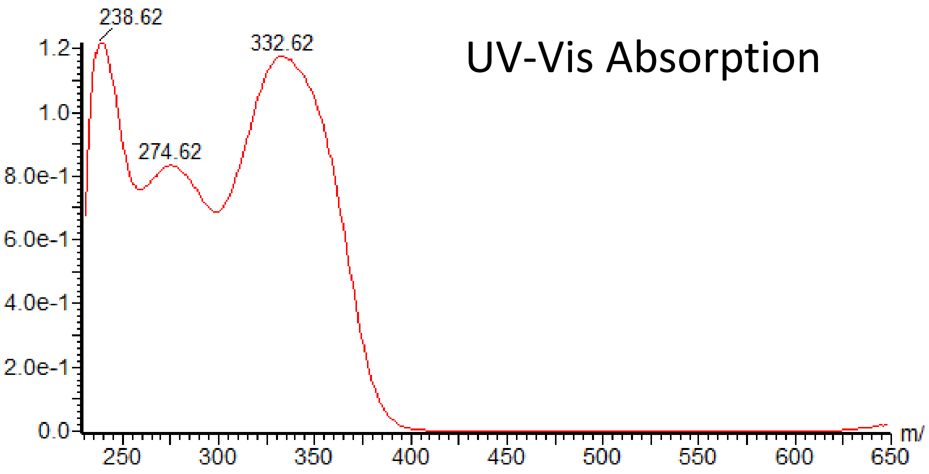

lupulone

RT: 4.19 – 4.37 minutes (wells A3-A4 Plate 2)

ESI –ve
(MW: 414.6)

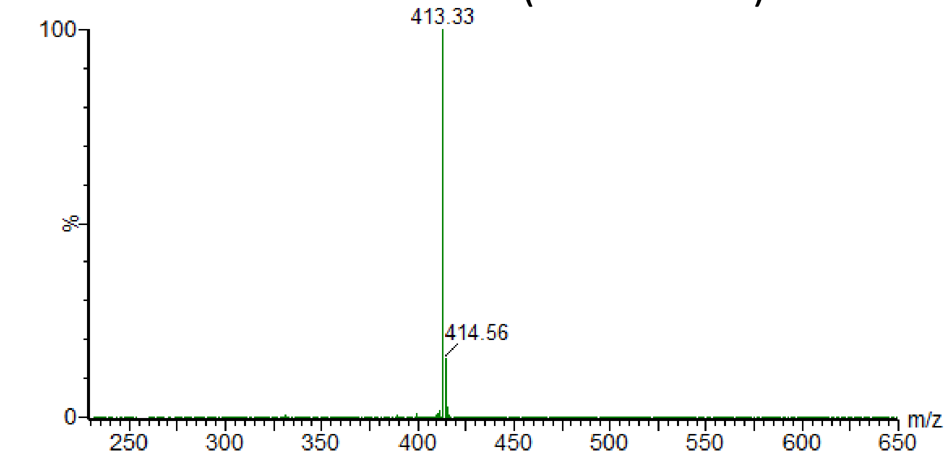

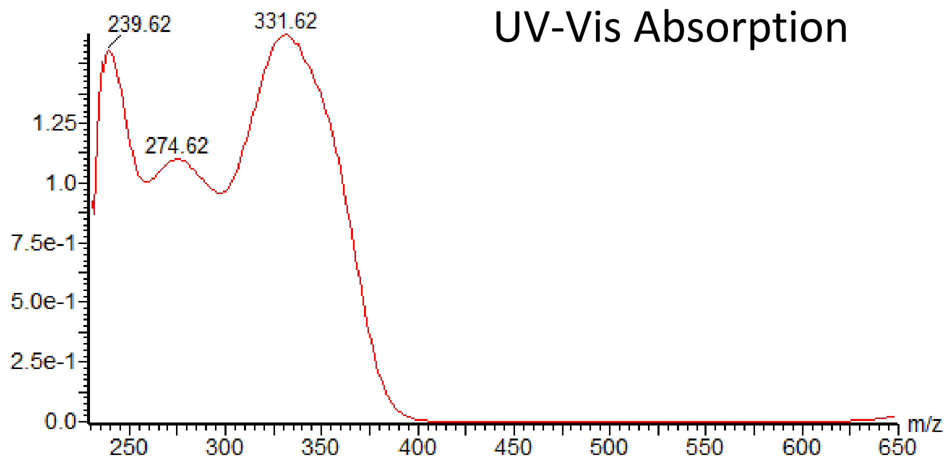

Supplementary Figure 1D: 5 second retention time window components beta acid enriched extract

adlupulone

RT: 4.37 – 4.53 minutes (wells A5-A6 Plate 2)

ESI –ve
(MW: 414.6)

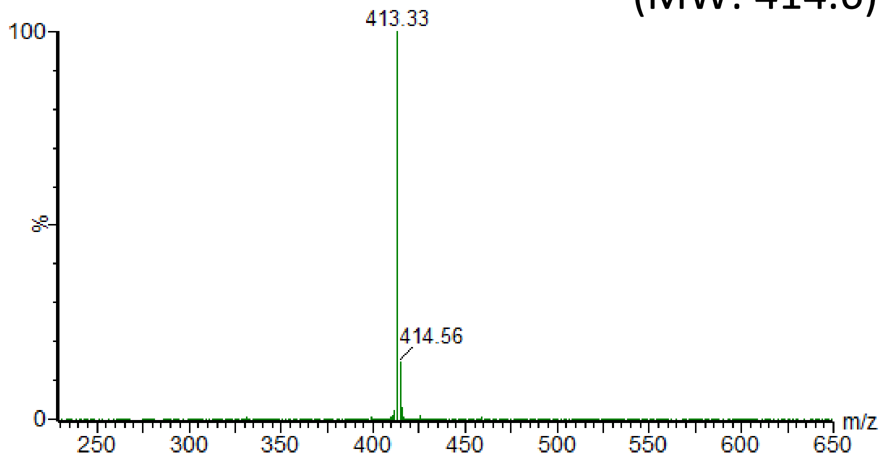

UV-Vis Absorption

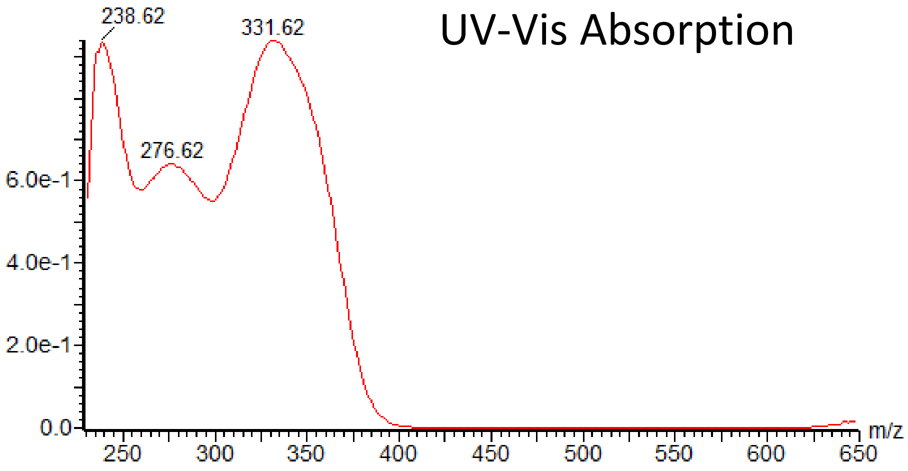

Supplement: Supplementary file 1 [file molecules-25-03677-s001.pdf]
